# Gaming-based program for internet gaming disorder: feasibility and preliminary outcomes of a structured camp program

**DOI:** 10.3389/fpsyt.2026.1825298

**Published:** 2026-06-09

**Authors:** Shanghao Yang, Bojie Zhou, Yingfeng Xie, Yueying Hu, Li Shi, Xuhui Zhou

**Affiliations:** 1 Department of Addiction Medicine, Hunan Institute of Mental Health, The School of Clinical Medicine, Hunan University of Chinese Medicine, Changsha, Hunan, China; 2 Department of Addiction Medicine, Hunan Institute of Mental Health, The Second People’s Hospital of Hunan Province (Brain Hospital of Hunan Province), Changsha, Hunan, China; 3 Fuzhou Neuropsychiatric Hospital Affiliated to Fujian Medical University, Fuzhou, China; 4 Neuropsychiatric Prevention and Treatment Hospital of Fuzhou Second General Hospital, Fuzhou, China; 5 College of Integrative Chinese and Western Medicine, Hunan University of Chinese Medicine, Changsha, China

**Keywords:** internet gaming disorder, adolescents, gaming-based program, camp program, craving, pilot evaluation

## Abstract

**Background:**

Although controlled trials support several psychosocial interventions for adolescent internet gaming disorder (IGD), short, highly structured residential camp formats remain underreported, particularly regarding feasibility, safety, and process data from routine service settings.

**Objective:**

To evaluate the feasibility, safety, and short-term entry-to-exit signals of a structured gaming-based camp program using retrospective, de-identified routinely collected service data, with a focus on implementation evidence for a brief, highly structured residential format.

**Methods:**

We conducted a single-group entry-to-exit evaluation of a 7-day structured camp in 12 adolescents aged 11–16 years clinically diagnosed with IGD by psychiatrists using DSM-5 criteria. Entry and exit assessments were organized hierarchically, with the Gaming Disorder Screening Scale (GDSS), the Game Addiction Scale–7 (GAS-7), and the Visual Analog Scale (VAS) craving score as primary outcomes; the Barratt Impulsiveness Scale–11 (BIS-11), Zung Self-Rating Depression Scale (SDS), Zung Self-Rating Anxiety Scale (SAS), Social Avoidance and Distress Scale (SADS), and Chinese version of the Interpersonal Reactivity Index (IRI-C) as supportive secondary outcomes; and camp residential counselor–rated Conners scores and the Stroop task as exploratory external-rating and objective complementary indicators, respectively. Wilcoxon signed-rank tests reported effect size r and Hodges–Lehmann (HL) median difference with 95% CI. Exploratory Spearman correlations examined baseline characteristics and change.

**Results:**

Completion was 100% (12/12) and attendance 98.6% (142/144), with no serious adverse events; two participants had a brief single-session interruption (coded as non-attendance for that session-person) but completed subsequent sessions and exit assessment. Primary outcomes decreased at exit: GDSS 50.00 (SD 8.32) to 28.67 (SD 8.98), p<0.001, r=0.88, HL −20.5 (95% CI −27.0 to −16.5); GAS-7 21.50 (SD 6.57) to 13.33 (SD 4.44), p=0.003, r=0.89, HL −8.5 (95% CI −12.5 to −6.0); VAS 4.25 (SD 2.63) to 2.75 (SD 2.05), p=0.012, r=0.85, HL −2.0 (95% CI −3.0 to −1.0). High-risk GDSS decreased from 66.7% to 0%, GAS-7 positivity from 58.3% to 8.3%, and moderate-to-severe craving from 58.3% to 25.0%. BIS-11, SDS, and IRI-C showed supportive short-term changes, and counselor-rated Conners scores showed exploratory external-rating changes, whereas SAS and SADS did not. Stroop reaction time decreased and accuracy increased in both conditions, while interference effects did not change significantly. Baseline PSQI correlated with change in VAS craving (ρ=0.767, p=0.004).

**Conclusions:**

This pilot program evaluation suggests that a short, highly structured gaming-based residential camp can be delivered feasibly and safely and may be associated with short-term reductions in IGD symptom severity and craving. Its main value is to provide early implementation and short-term signal data for a service-based camp format that should be tested in subsequent controlled studies with follow-up.

## 1 Introduction

Internet gaming disorder in adolescents has been regarded as a behavioral addiction of clear public health and clinical significance. Its core features involve impaired control over gaming behavior, assigning excessive priority to gaming activities, and persistence or escalation despite negative consequences, leading to significant functional impairment and distress. Differences in diagnostic frameworks and measurement tools have contributed to variation in prevalence estimates and cross-study comparability (1), underscoring the need for intervention studies to define eligibility and outcome priorities clearly (2). This is particularly relevant because adolescent IGD is not simply a matter of excessive gaming time, but a multidimensional clinical presentation involving core gaming symptoms and related behavioral, emotional, and interpersonal domains. In this broader clinical context, craving, impaired control, affective distress, and social functioning are particularly relevant to adolescent IGD and to the selection of intervention targets and supportive assessment domains. Prior studies have linked IGD symptoms with anxiety, psychological distress, externalizing problems, reward-processing bias, impulsivity, and impaired inhibitory control (3–5). Family and parental factors may also shape adolescents’ symptom trajectories, supporting the inclusion of family-context and other-rater perspectives in intervention and evaluation frameworks (6).

Existing intervention research suggests that psychosocial interventions can reduce gaming-disorder symptoms, but the evidence base remains heterogeneous in setting, intervention dose, outcome selection, and follow-up duration (7, 8). Most controlled studies have examined school-based, family-focused, or group-based formats (9–11), whereas brief residential service models have been less frequently documented (12). This distinction matters because a residential camp is not simply a shorter version of outpatient therapy; it raises different implementation questions regarding deliverability, safety, engagement, supervision, process capture, and short-term response during a temporarily controlled living environment. Therefore, before such programs can be tested as efficacy interventions, their feasibility, safety, acceptability, and short-term outcome signals need to be described more clearly.

A brief residential camp may be useful to study because it temporarily changes the treatment context. By removing access to internet-enabled devices, imposing a structured daily routine, and placing adolescents in repeated staff-supervised group tasks, a camp format can reduce immediate gaming cues while creating opportunities for offline reinforcement, peer interaction, behavioral rehearsal, and direct observation of engagement and dysregulation. These features are especially relevant for adolescents with IGD, whose usual environments often contain persistent gaming cues, peer reinforcement, and limited opportunities to practice non-gaming alternatives. At the same time, the same features make camp programs complex and difficult to interpret: observed changes may reflect cue restriction, structured routines, counselor support, group dynamics, or specific therapeutic activities. This complexity makes implementation-oriented pilot evaluation necessary before stronger claims about efficacy can be made.

The camp was therefore guided by a focused psychosocial model rather than by a single technique. Drawing on cognitive–behavioral principles for behavioral addictions and process models such as I-PACE (13–15), we conceptualized adolescent IGD as a reinforced behavioral pattern maintained by craving, impaired control, emotion-regulation motives, maladaptive gaming-related cognitions, and weak offline sources of mastery and social reward. The camp attempted to address these processes through temporary cue reduction, structured routines, non-digital gamified tasks, emotion-regulation rehearsal, cooperative peer activities, and relapse-prevention planning.

This change model also guided the selection of assessment domains. Core IGD symptom severity and craving were treated as proximal outcome signals because they most directly reflected the behavioral pattern and craving-driven response chain targeted by the camp. Impulsivity, affective distress, social avoidance, interpersonal responsiveness, and counselor-rated behavioral dysregulation were included as supportive or exploratory domains because they corresponded to the camp’s behavioral-control, emotion-regulation, and peer-interaction components. Sleep quality and family functioning were assessed only as baseline contextual domains because they may shape craving, emotion regulation, daily routines, and response heterogeneity, but were not expected to show interpretable change within seven days. Detailed instrument-level rationale, scoring, interpretation, and analytic boundaries are provided in the Methods and **Supplementary Table 13**.

Against this background, the present study was designed as a retrospective, de-identified, single-group pilot evaluation of a 7-day structured residential camp delivered as routine service for adolescents with IGD. The primary aim was to document feasibility and safety, including completion, attendance, implementation deviations, and adverse events. A secondary aim was to describe acceptability, counselor-observed process indicators, and short-term entry-to-exit changes in prespecified core outcomes, with IGD symptom severity and craving treated as the main outcome signals. Broader emotional, behavioral, interpersonal, external-rating, and objective task measures were analyzed as supportive or exploratory indicators, while baseline sleep and family-functioning measures were used only for contextual characterization and hypothesis generation. The study was not designed to establish treatment efficacy, but to provide implementation and short-term signal data to guide subsequent controlled studies with follow-up.

## 2 Methods

### 2.1 Study design and setting

This retrospective analysis used routinely collected, de-identified service data from a single-group entry-to-exit program evaluation. A 7-day closed residential camp was delivered as routine clinical/rehabilitation services at Ziyang District Psychiatric Hospital, Yiyang, Hunan, China from August 2 to August 8, 2024. Ongoing treatments continued under routine procedures; medication details were not extracted.

### 2.2 Ethics and informed consent

The Ethics Committee of the Second People’s Hospital of Hunan Province approved the retrospective secondary analysis and publication of de-identified service data (approval no. 2025K087) under an inter-institutional reliance arrangement for the program conducted at Ziyang District Psychiatric Hospital. Written guardian consent and adolescent assent were obtained at enrollment for participation and routine assessments, including permission for research use and publication of de-identified data. Data were de-identified and stored with restricted access, and predefined safety procedures were used for group conflict or emotional escalation.

### 2.3 Participants

#### 2.3.1 Recruitment and diagnostic confirmation

12 participants were referred from psychiatric outpatient and inpatient services. Internet gaming disorder was confirmed by qualified psychiatrists using DSM-5 criteria. Assessments were completed on the day of entry and the day of exit, and all enrolled participants completed the program and both assessments (see **Supplementary Figure 1**).

#### 2.3.2 Inclusion and exclusion criteria

Eligible adolescents were aged 10–17 years, met DSM-5 criteria for Internet gaming disorder, had engaged in online gaming within the past year, and could complete the 7-day closed residential camp. Exclusion criteria were current or lifetime substance use disorder, inability to obtain guardian consent/adolescent assent, or any acute/unstable psychiatric or medical condition that could compromise safe participation (e.g., acute psychosis/mania, severe cognitive impairment, acute suicidality or significant self-harm risk). Psychotic disorders were eligible only if clinically stable at entry and judged suitable by a psychiatrist.

#### 2.3.3 Baseline demographic and clinical variables

Baseline data from structured questionnaires and clinical interviews included demographics, treatment setting, age at gaming initiation, comorbidities, and indicators of internet/gaming intensity. Baseline self-reported gaming time could be 0 because of temporary pre-camp restrictions.

### 2.4 Intervention

The intervention was a 7-day structured residential camp comprising 12 standardized sessions and was guided by a prespecified psychosocial framework in which rule-governed, gamified therapeutic tasks served as the delivery format rather than the treatment target itself. The framework combined cognitive–behavioral and motivational elements with emotion-regulation practice, alternative offline reinforcement, social-skills rehearsal, self-reflection, and relapse-prevention planning to reduce cue-driven and emotion-avoidant gaming, strengthen self-monitoring and delay of gratification, restore real-world reward and peer affiliation, and consolidate coping strategies for high-risk situations. Participants had no access to internet-enabled devices throughout the camp, with one supervised nightly voice call arranged via the counselor’s phone. The curriculum followed a fixed schedule and was implemented uniformly, supported by standardized lesson plans, staff training, and structured recording forms. Session-level therapeutic targets and gamification techniques are detailed in **Supplementary Table 1**. For example, one session used a “boss battle” metaphor to help participants identify emotional triggers and rehearse coping responses, whereas another session used a “little monster” metaphor to externalize craving and guide pause-and-choose responses to gaming urges. Taken together, the camp should be understood as an integrated psychosocial intervention rather than as a set of stand-alone activities. The camp was delivered in a mixed-age format without formal age-stratified modules, and camp residential counselors provided continuous one-to-one accompaniment, supported task participation, enforced rules, and documented behavioral observations.

The intervention operationalized this cognitive–behavioral and behavioral-addiction change model through five linked therapeutic processes. First, cue-context management was implemented by removing access to smartphones, internet-enabled devices, and online gaming throughout the residential period. Second, alternative offline reinforcement was provided through structured physical, art-based, exploratory, and cooperative tasks that generated immediate feedback, mastery experiences, and peer recognition outside gaming contexts. Third, behavioral-control training was embedded in rule-governed tasks requiring turn-taking, delayed responding, task persistence, adherence to group rules, and “pause-and-choose” responses to craving or impulsive urges. Fourth, emotion-regulation and cognitive–behavioral rehearsal were delivered through activities focused on identifying negative emotions, externalizing craving, mapping gaming-related triggers, generating coping responses, and reflecting on alternative behaviors. Fifth, social-learning and relapse-prevention processes were incorporated through group cooperation, role-play, peer feedback, communication practice, and development of a post-camp maintenance plan.

Gamification was used only as a non-digital, staff-structured delivery strategy and did not involve online games, competitive video-game play, or screen-based rewards. Because the program combined cue reduction, structured routines, alternative reinforcement, skill rehearsal, and peer-based learning, it was not designed to isolate the independent effect of any single component; the conceptual mapping is summarized in **Supplementary Table 12**.

#### 2.4.1 Assessment rationale, score direction, and outcome definitions

The assessment battery was selected to map onto the theory-guided maintenance and change model described above, rather than to treat all measured domains as equivalent outcome endpoints. The pre–post outcomes were limited to domains that were plausibly relevant to short-term changes in craving, behavioral control, affective distress, social engagement, interpersonal responsiveness, and observable behavioral regulation during the 7-day residential program. At the same time, given the brief duration and single-group design, secondary and exploratory measures were interpreted as supportive domain-level indicators rather than as independent evidence of treatment benefit. For measures that may partly reflect more stable individual characteristics, particularly BIS-11 impulsivity and IRI-C interpersonal responsiveness, entry-to-exit changes were interpreted as short-term questionnaire-based shifts and supportive signals rather than as evidence of durable trait modification.

GDSS, GAS-7, and VAS craving were selected as primary outcomes because they directly assessed core Internet gaming disorder severity and proximal craving, which were the most immediate targets of cue-context management, craving-coping rehearsal, and non-gaming alternative reinforcement (13–15). BIS-11 was included to assess impulsivity because the camp repeatedly trained rule adherence, delay of gratification, task persistence, and pause-and-choose responses to urges (5, 13–15). SDS and SAS were included to assess depressive and anxiety symptoms because emotion-avoidant gaming and negative-affect regulation were part of the hypothesized maintenance model, and the curriculum included emotion identification, coping rehearsal, and behavioral activation (3, 4, 15, 16). SADS was included to assess social avoidance and distress because the program aimed to increase offline social participation through cooperative tasks, role-play, and peer interaction (23, 34). IRI-C was included to assess interpersonal responsiveness because group cooperation, perspective-taking, feedback, and role rehearsal were expected to engage interpersonal processes, although these changes were interpreted cautiously over a 7-day interval (6, 10, 24, 25, 34). Camp residential counselor-rated Conners scores were included as exploratory structured external observations of attention problems, impulsivity/hyperactivity, task persistence, and behavioral regulation in the residential context, because these domains were observable during repeated rule-governed tasks, group activities, meals, and daily routines, and were directly aligned with the camp’s behavioral-control targets. They were intended to complement self-report questionnaires and session-specific counselor ratings, not to provide a diagnostic ADHD assessment or a standard school-based teacher rating (26, 39). PSQI and FACES II-CV were assessed at baseline only because sleep quality and family functioning were conceptualized as contextual vulnerability or response-modifying factors, rather than as short-interval outcomes directly targeted by the 7-day camp (6, 10, 41, 42).

Detailed information on each assessment instrument, including the construct assessed, timing, number of items, response scale, scoring range, direction of interpretation, criteria used in this study, rationale for selection, analytic role, and interpretive boundary, is provided in **Supplementary Table 13**.

All measures used Chinese or Chinese-adapted versions and were scored according to manuals, including required reverse scoring, with analyses based on total or standard scores as appropriate. For readability, their construct coverage and interpretive roles are defined centrally in this subsection: GDSS and GAS-7 indexed Internet gaming disorder symptom severity, VAS indexed craving on a 0–10 scale, BIS-11 indexed impulsivity, SDS and SAS indexed depressive and anxiety symptoms, SADS indexed social avoidance/distress, IRI-C indexed interpersonal responsiveness, and Conners provided an exploratory external rating of behavioral dysregulation in the camp context. Higher scores indicated greater symptom burden or dysregulation unless otherwise specified; higher IRI-C total scores were interpreted cautiously as greater self-reported interpersonal responsiveness, whereas higher session-level counselor observation ratings indicated better in-camp functioning. The assessment set was prespecified hierarchically according to proximity to the camp’s intended targets and the plausibility of interpretable change over a 7-day interval.

Primary outcomes were entry-to-exit changes in Gaming Disorder Screening Scale (GDSS) total score (17), Game Addiction Scale–7 (GAS-7) total score (18), and Visual Analog Scale (VAS) craving score (19) because these measures most directly indexed core IGD symptom severity and proximal craving and were prespecified as the main targets expected to be most responsive over the brief camp interval. Given the longer clinical anchor windows of some symptom measures, observed changes were interpreted as short-term within-person shifts rather than as definitive evidence of durable clinical remission.

Secondary questionnaire outcomes were entry-to-exit changes in Barratt Impulsiveness Scale–11 (BIS-11) total score (20), Zung Self-Rating Depression Scale (SDS) standard score (21), Zung Self-Rating Anxiety Scale (SAS) standard score (22), Social Avoidance and Distress Scale (SADS) total score (23), and Chinese version of the Interpersonal Reactivity Index (IRI-C) total score (24, 25). These measures were selected to characterize broader domains commonly linked to adolescent IGD, including impulsivity, affective distress, social discomfort, and interpersonal functioning, and were treated as supportive rather than co-primary outcomes because they were not expected to change uniformly over a 7-day interval. Camp residential counselor–rated Conners scores (26) were retained as exploratory external ratings of observable behavioral dysregulation in the camp context rather than as diagnostic indicators or core symptom endpoints.

Pittsburgh Sleep Quality Index (PSQI) (27) and Family Adaptability and Cohesion Evaluation Scales II, Chinese Version (FACES II-CV) (28, 29) were assessed at camp entry only to characterize baseline sleep-related burden and family-context features that may shape craving, affective regulation, behavioral control, daily routines, and response heterogeneity (6, 10, 41, 42). They were not repeated at camp exit because sleep quality and family functioning were conceptualized as contextual vulnerability or response-modifying domains rather than as primary targets expected to show interpretable change over the 7-day camp interval. Accordingly, PSQI and FACES II-CV were used only for baseline description and exploratory association analyses and were excluded from the pre–post outcome set (**Supplementary Table 4**).

#### 2.4.2 Objective task: stroop task

An exploratory computerized Stroop task was administered at entry and exit to provide an objective complementary indicator of inhibitory control using game-related and neutral words, because inhibitory-control dysfunction is theoretically relevant to IGD but was not treated as a core short-interval outcome endpoint in this 7-day program. Reaction time and accuracy were recorded, and interference effects were defined as game-related minus neutral for both outcomes. Analyses used correct-response trials and prespecified reaction-time cleaning rules (e.g., <200 ms or >3000 ms; within-participant extremes beyond ±3 SD), with handling of insufficient usable trials documented in the Results.

#### 2.4.3 Feasibility, implementation fidelity, safety, and acceptability

Feasibility and fidelity were assessed using completion and attendance (session-person counts), implementation-deviation logs, and documented group conflict incidents (**Supplementary Table 2**). Safety was monitored throughout the camp, with serious adverse events predefined and recorded (**Supplementary Table 3**). Acceptability was assessed via counselor session observation ratings and participant exit satisfaction ratings using prespecified scoring rules (**Supplementary Tables 9** and **S10**). Conners ratings and counselor session observation ratings served different purposes. Conners provided a structured pre–post external rating of broader observable behavioral dysregulation in the residential camp context, including attention, impulsivity/hyperactivity, task persistence, and behavioral regulation. In contrast, counselor session observation ratings were process measures completed after each session to capture participation quality, cooperation, task completion, communication, engagement, and emotion regulation during specific camp activities.

#### 2.4.4 Clinical stratification and responder definitions

Prespecified thresholds were used descriptively for stratification and responder analyses and are summarized in **Supplementary Table 6** to centralize the interpretive rules for the clinically anchored measures. Here, “prespecified” refers to rules defined in the service evaluation protocol or scoring plan before statistical analysis, rather than to prospective trial registration. In the main text, we report core thresholds for GDSS, GAS-7, VAS, SDS, and SAS; all other thresholds and operational definitions are provided in **Supplementary Table 6**. Conners reference cutoffs were used descriptively only and were not used for diagnostic classification. No formal responder definition was prespecified for Conners or Stroop because both were conceptualized as exploratory supportive indicators rather than as primary or key secondary treatment endpoints.

### 2.5 Data collection and quality control

Questionnaire assessments were completed on the day of camp entry and on the day of camp exit. Counselor observation ratings were completed independently after each session, and satisfaction ratings were collected at camp exit. All data were de-identified and independently double-entered by two staff members; discrepancies were resolved by referring to the original source forms, with verification and consistency checks. No missing paired entry-to-exit questionnaire data were observed for the outcomes analyzed. The program team provided standardized training and centralized management of program delivery and assessment procedures to minimize implementation variability.

### 2.6 Statistical analysis

Analyses were performed in SPSS 26.0, with additional scripts for Hodges–Lehmann estimates with 95% confidence intervals and figure generation. Continuous variables are summarized as mean ± SD and median (IQR); categorical variables as n (%). Entry-to-exit comparisons used two-sided Wilcoxon signed-rank tests, reporting exact p values as primary inference and asymptotic p values alongside Z for effect-size computation. Effect size r was calculated as |Z| divided by the square root of the effective number of non-zero paired differences. Hodges–Lehmann estimates and 95% confidence intervals and standardized paired change d_z were reported as prespecified. Effect sizes and confidence intervals were reported to aid interpretation, but estimates are expected to be unstable in this small pilot sample. Primary outcomes constituted the main evaluation focus. Secondary questionnaire outcomes were treated as supportive domain-level indicators, whereas Conners ratings, Stroop variables, and association analyses were treated as exploratory; accordingly, no multiple-comparison correction was applied outside the primary outcome set. Exploratory associations used Spearman correlations; two-sided p < 0.05 was considered statistically significant, and 0.05–0.10 was considered a trend and denoted by † in **Supplementary Table 11**.

## 3 Results

### 3.1 Participants, feasibility, and data completeness

Twelve adolescents with Internet gaming disorder participated in the camp program and all completed the 7-day camp and the camp-exit assessment (completion, 100%). All 12 sessions were delivered as planned (no cancellations or substantive shortening). Attendance was 98.6% (142/144 session-person counts); 10 participants completed all sessions, and 2 had a brief session interruption (temporary step-out) during the “Defeating Negative Emotions” session. Per the prespecified attendance coding rule, this interruption was coded as non-attendance for that session-person (2/144), but both participants returned and completed the remainder of the session, attended all subsequent sessions, and completed the camp-exit assessment. One interpersonal conflict occurred on day 3 and was de-escalated on site by a psychiatrist without discontinuation. No session-level delivery deviations affecting the core intervention content were observed. Data completeness was 100% for all paired entry-to-exit outcomes included in this analysis (no missing paired data); PSQI and FACES II-CV were assessed at baseline only by design and were therefore used for baseline characterization and exploratory baseline-to-change analyses rather than for pre–post comparisons. Feasibility and fidelity metrics are summarized in **Supplementary Table 2**.

### 3.2 Baseline characteristics

Baseline characteristics are summarized in **Table 1**, with expanded psychosocial profiles in **Supplementary Table 4**. These baseline-only measures were included to describe the clinical and family-context background of the enrolled sample and to support exploratory interpretation of heterogeneity in short-term change, not to imply that sleep or family functioning was evaluated as an immediate treatment outcome. The sample comprised 12 adolescents (age 14.25 ± 1.29 years; 66.7% male) from outpatient (66.7%) and inpatient (33.3%) settings. The mean age at onset of online gaming was 11.50 ± 2.07 years. Baseline digital exposure was substantial (internet use 6.79 ± 4.22 h/day; gaming 4.67 ± 3.31 h/day). Poor sleep quality was common (PSQI 8.75 ± 3.72; 66.7% > 5), and family functioning on FACES II-CV indicated lower actual than ideal functioning.

**Table 1:** Baseline characteristics of participants (N = 12).

Variable	Value
Age (years, Mean ± SD [range])	14.25 ± 1.29 [11–16]
Gender (male/female, n [%])	8 (66.7%) / 4 (33.3%)
Treatment setting (outpatient/inpatient, n [%])	8 (66.7%) / 4 (33.3%)
Age of onset of online gaming (years, Mean ± SD [range])	11.50 ± 2.07 [8–15]
Comorbidities (n [%])	None: 7 (58.3%); Major depressive episode: 4 (33.3%); Schizophrenia: 1 (8.3%)
Daily internet use (hours, Mean ± SD [range])	6.79 ± 4.22 [0.5–12]
Weekly internet use (hours, Mean ± SD [range])	35.75 ± 28.07 [2–84]
Weekly internet use days (days, Mean ± SD [range])	5.50 ± 2.20 [2–7]
Daily online gaming (hours, Mean ± SD [range])	4.67 ± 3.31 [0–11]
Weekly online gaming (hours, Mean ± SD [range])	23.58 ± 17.52 [2–65]
Weekly online gaming days (days, Mean ± SD [range])	5.08 ± 2.23 [1–7]

Values are presented as mean ± SD with range in brackets, or n/N (%), as appropriate. Comorbidities were clinician-diagnosed at baseline. Baseline daily online gaming time could be 0 because temporary restrictions had already been implemented during the week before camp entry (e.g., inpatient supervision or family-imposed limits), although DSM-5 IGD diagnosis reflected a pre-existing course of persistent gaming-related impairment. The participant with schizophrenia was clinically stable at camp entry and was cleared for participation by a psychiatrist.

### 3.3 Primary outcomes

**Figure 1:**
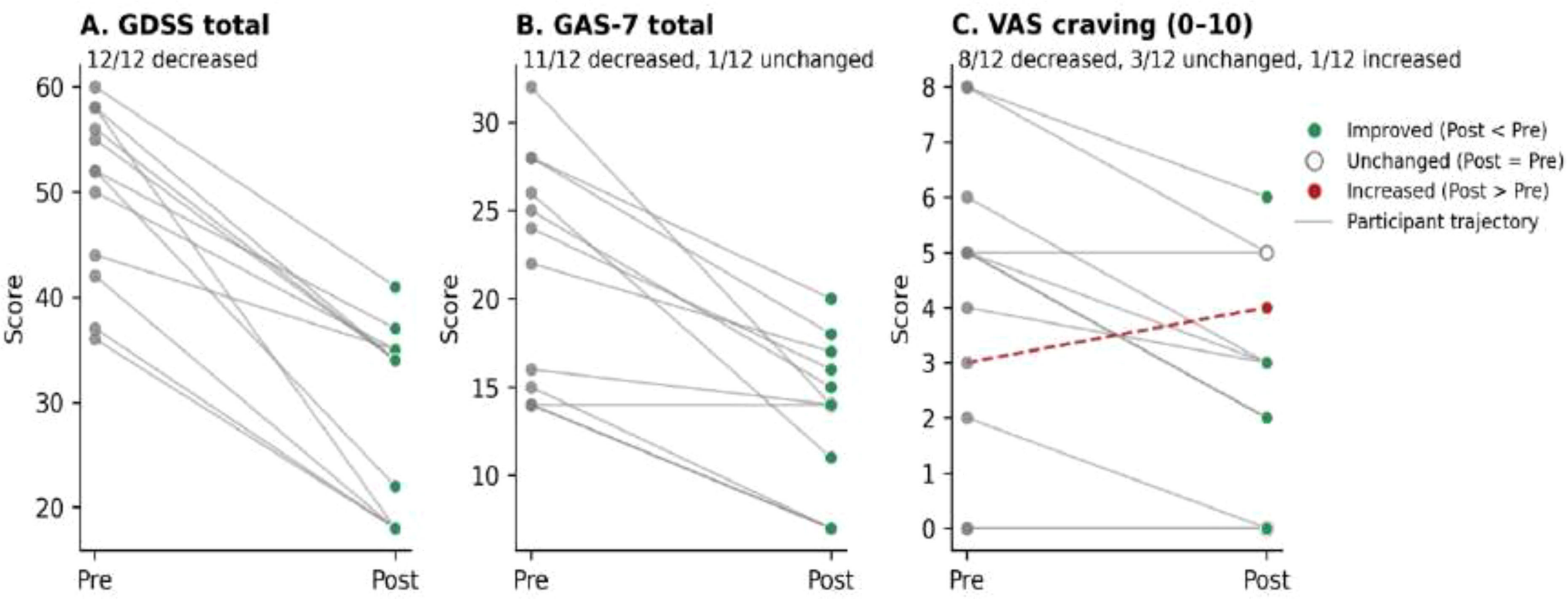
Individual trajectories of primary outcomes (n=12). Lines denote participant-level paired scores (Pre vs Post). Pre scores are shown in gray. Post-camp markers indicate direction of change: green filled circles indicate lower scores at camp exit (Post<Pre), open circles indicate no change (Post=Pre), and red filled circles indicate worsening (Post>Pre). Higher scores indicate greater IGD severity (GDSS, GAS-7) or craving (VAS). Δ=Post−Pre.

All primary outcomes showed reductions from camp entry to camp exit (**Table 2**; **Supplementary Table 5**; **Figure 1**). GDSS total decreased markedly (Δ=Post–Pre: −21.33 ± 7.94; HL=−20.5 [95% CI −27.0, −16.5]; exact p<0.001; r=0.88; d^z^=2.69), with decreases in 12/12 participants. GAS-7 total also decreased (Δ=−8.17 ± 4.93; HL=−8.5 [−12.5, −6.0]; exact p=0.003; r=0.89; d^z^=1.66), with 11/12 decreased and 1/12 unchanged. VAS craving (0–10) decreased overall (Δ=−1.50 ± 1.45; HL=−2.0 [−3.0, −1.0]; exact p=0.012; r=0.85; d^z^=1.03), with paired trajectories showing 8/12 decreased, 3/12 unchanged, and 1/12 increased.

**Table 2:** Primary outcomes at camp entry and camp exit (n=12).

Outcome	Camp entry (Pre) Mean ± SD; Median (IQR) [range]	Camp exit (Post) Mean ± SD; Median (IQR) [range]	Δ (Post−Pre) Mean ± SD [range]	HL median diff	95% CI (HL)	Wilcoxon exact p (two-sided)	Effect size r	Cohen’s dz
GDSS total	50.00 ± 8.32 52.0 (43.5–56.5) [36–60]	28.67 ± 8.98 34.0 (18.0–35.0) [18–41]	-21.33 ± 7.94 [-40, -9]	-20.5	-27.0 to -16.5	<0.001	0.88	2.69
GAS-7 total	21.50 ± 6.57 23.0 (14.8–26.5) [14–32]	13.33 ± 4.44 14.0 (10.0–16.2) [7–20]	-8.17 ± 4.93 [-18, 0]	-8.5	-12.5 to -6.0	0.003	0.89	1.66
VAS craving (0–10)	4.25 ± 2.63 5.0 (2.8–5.2) [0–8]	2.75 ± 2.05 3.0 (1.5–4.2) [0–6]	-1.50 ± 1.45 [-3, 1]	-2.0	-3.0 to -1.0	0.012	0.85	1.03

Δ=Post−Pre. HL denotes the Hodges–Lehmann estimate of the median paired difference. 95% CIs were obtained using the distribution-based method associated with the Wilcoxon signed-rank procedure. Wilcoxon signed-rank tests are two-sided; exact p values are reported. Effect size r=|Z|/√n_e_ff, where effective n is the number of non-zero paired differences. Cohen’s d^z^=|mean(Δ)|/SD_Δ.

Prespecified threshold-based outcomes are summarized in **Supplementary Table 6** (and **Supplementary Figure 2**). The proportion meeting the GDSS high-risk threshold (≥47) decreased from 66.7% to 0%, and 75.0% met the responder criterion used in this analysis (≥50% reduction in GDSS total). GAS-7 positivity decreased from 58.3% to 8.3% under the polythetic rule (6 positive-to-negative conversions) and from 16.7% to 0% under the monothetic rule. For craving, moderate-to-severe levels (VAS≥5) decreased from 58.3% to 25.0%, while the proportion reporting no craving (VAS = 0) increased from 16.7% to 25.0%. The single increase was small (from 3 to 4 on the 0–10 VAS) and occurred despite the overall downward group trend, indicating individual heterogeneity in short-term craving response.

### 3.4 Secondary outcomes

**Figure 2:**
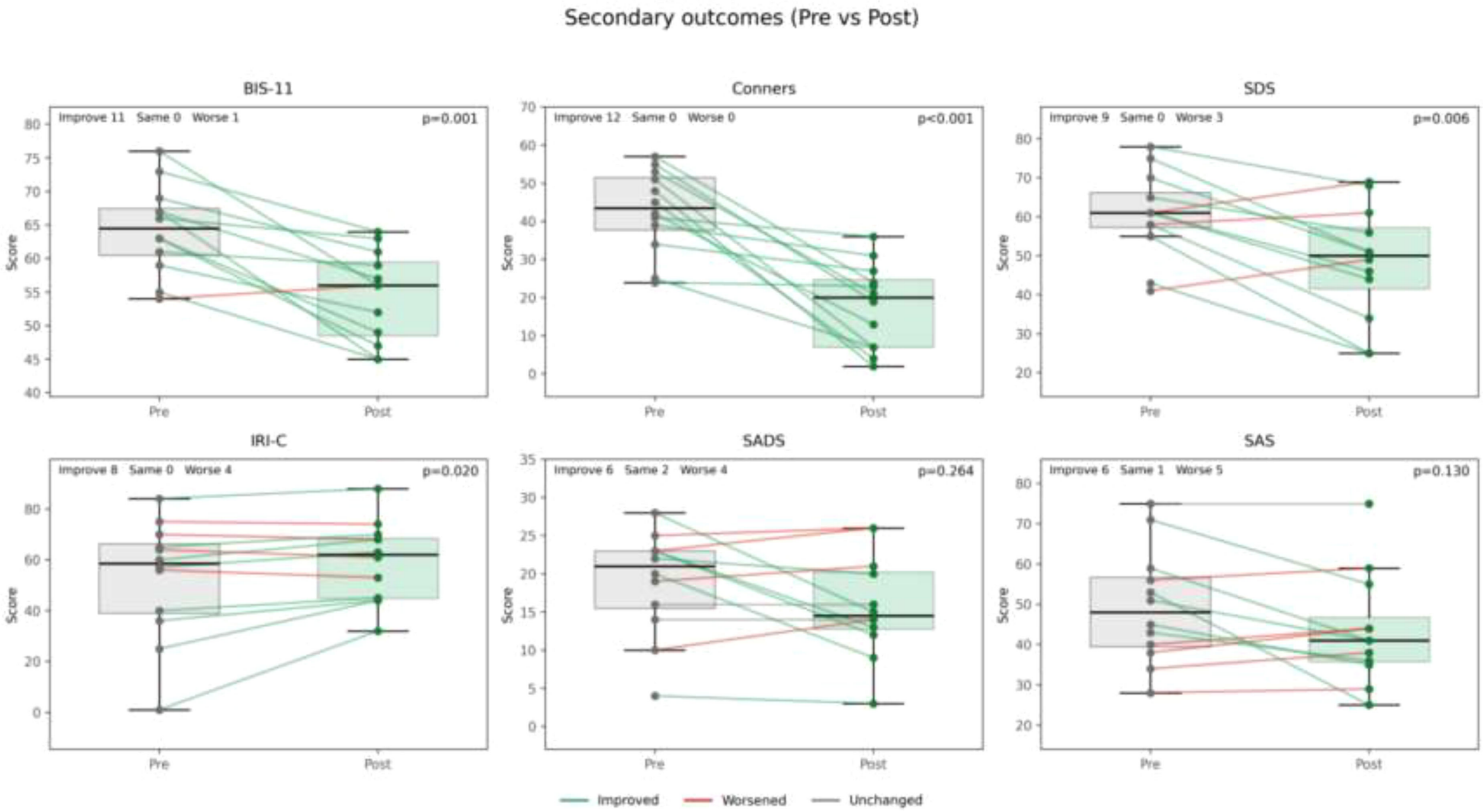
Secondary outcomes from camp entry to camp exit (n=12). Boxes show the interquartile range with the median line; whiskers indicate the range. Lines denote participant-level paired scores. p values are from two-sided Wilcoxon signed-rank tests (exact p) as reported in **Table 3**. For BIS-11, Conners, SDS, SAS, and SADS, lower post-camp scores indicate a direction consistent with reduced symptom burden or dysregulation; for IRI-C, higher post-camp scores indicate an increase in self-reported interpersonal responsiveness. Δ=Post−Pre.

Supportive secondary questionnaire outcomes and exploratory camp residential counselor-rated Conners scores are summarized in **Table 3** (exact p as the primary basis for inference), with Z and asymptotic p values provided in **Supplementary Table 7**; individual paired changes are visualized in **Figure 2**. Questionnaire-based domain measures and camp residential counselor–rated Conners scores should be interpreted as supportive or exploratory indicators rather than as evidence of comparable responsiveness across all non-primary domains. Statistically significant entry-to-exit changes were observed in impulsivity and counselor-rated observable behavioral dysregulation, with reductions in BIS-11 total (exact p=0.001) and camp residential counselor–rated Conners total (exact p<0.001). Depressive symptoms also decreased (SDS standard score, exact p=0.006), whereas anxiety did not show a statistically significant change (SAS standard score, exact p=0.130). Social avoidance/distress did not change significantly (SADS total, exact p=0.264). IRI-C total scores increased (exact p=0.020), interpreted cautiously as a short-term shift in self-reported interpersonal responsiveness. Prespecified threshold-based rates are reported in **Supplementary Figure 2**.

**Table 3:** Supportive secondary and exploratory external-rating outcomes at camp entry and camp exit (n=12).

Outcome	Pre (mean ± SD; median[IQR] [range])	Post (mean ± SD; median[IQR] [range])	Δ (Post−Pre) mean ± SD [range]	HL median diff	95% CI (HL)	Wilcoxon exact p (two-sided)	Effect size r	Cohen’s dz
BIS-11 total	64.42 ± 6.65; 64.50 (60.50, 67.50) [54.00, 76.00]	54.50 ± 6.80; 56.00 (48.50, 59.50) [45.00, 64.00]	-9.92 ± 7.18 [-22.00, 2.00]	-9.50	-15.00 to -5.00	0.001	0.851	1.381
Conners (camp residential counselor–rated) total	42.83 ± 10.95; 43.50 (37.75, 51.50) [24.00, 57.00]	17.83 ± 11.13; 20.00 (7.00, 24.75) [2.00, 36.00]	-25.00 ± 16.37 [-46.00, -1.00]	-24.75	-37.50 to -13.00	<0.001	0.883	1.528
SDS (standard score)	60.42 ± 11.10; 61.25 (56.88, 66.25) [41.25, 77.50]	48.23 ± 14.64; 50.00 (41.25, 57.50) [25.00, 68.75]	-12.19 ± 12.57 [-30.00, 7.50]	-13.13	-20.63 to -3.75	0.006	0.749	0.970
SAS (standard score)	49.27 ± 14.46; 48.12 (39.38, 56.88) [27.50, 75.00]	43.44 ± 13.78; 41.25 (35.94, 46.56) [25.00, 75.00]	-5.83 ± 10.58 [-27.50, 6.25]	-6.25	-13.75 to 2.50	0.130	0.471	0.551
SADS total	18.92 ± 6.84; 21.00 (15.50, 23.00) [4.00, 28.00]	15.75 ± 6.69; 14.50 (12.75, 20.25) [3.00, 26.00]	-3.17 ± 6.22 [-13.00, 4.00]	-4.00	-10.50 to 1.50	0.264	0.372	0.509
IRI-C total	52.75 ± 23.39; 58.50 (39.00, 66.25) [1.00, 84.00]	59.17 ± 15.90; 62.00 (44.75, 68.50) [32.00, 88.00]	6.42 ± 9.91 [-3.00, 31.00]	5.00	1.00 to 13.50	0.020	0.658	0.647

Δ=Post−Pre. HL denotes the Hodges–Lehmann estimate of the median paired difference. 95% CIs were obtained using the distribution-based method associated with the Wilcoxon signed-rank procedure. Wilcoxon signed-rank tests are two-sided; exact p values are reported. Effect size r=|Z|/√n_e_ff, where effective n is the number of non-zero paired differences. Cohen’s d^z^=|mean(Δ)|/SD_Δ.

### 3.5 Stroop task performance

**Figure 3:**
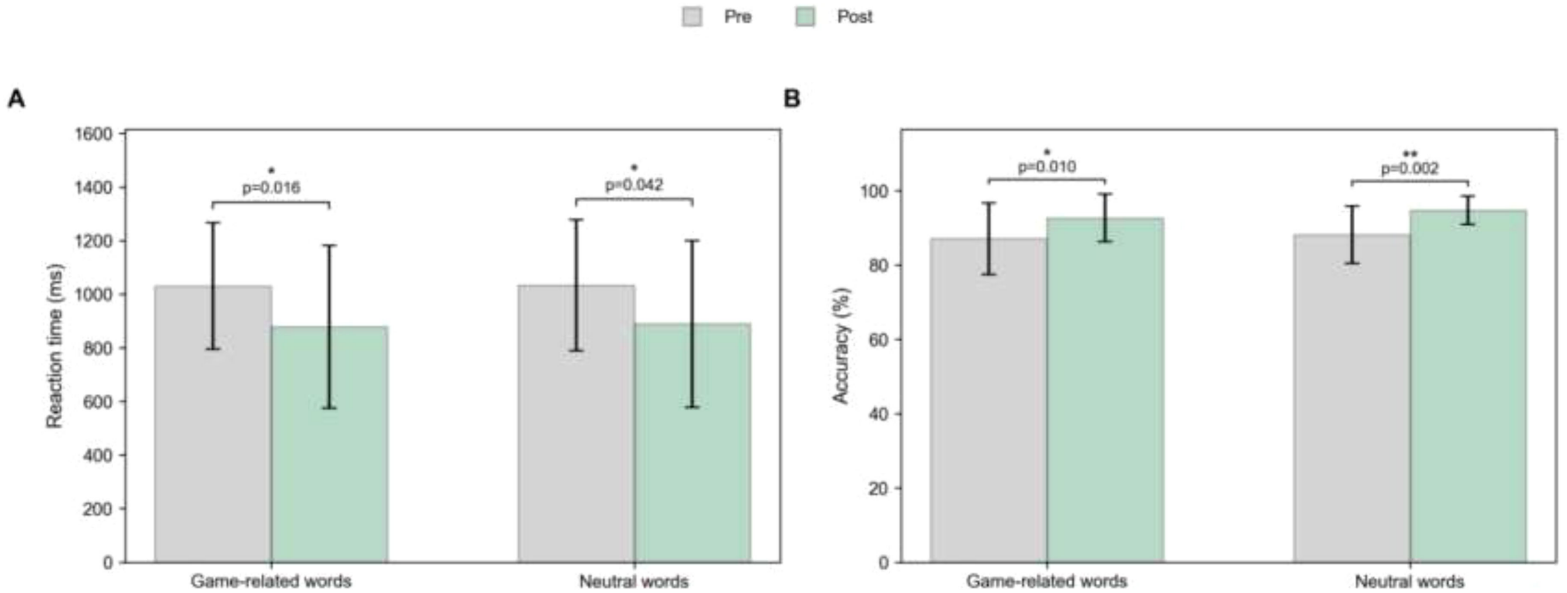
Stroop task performance at camp entry and camp exit (n=12). Bars show mean reaction time and accuracy by condition, and error bars indicate standard deviation. Accuracy is displayed as percentages (i.e., 100 × proportion) in this figure, while **Table 4** reports proportions. Exact p values from two-sided Wilcoxon signed-rank tests are shown above brackets. Interference indices are reported in **Table 4**. Δ=Post−Pre.

Stroop task outcomes are summarized in **Table 4** and **Figure 3**, with Z and asymptotic p values provided in **Supplementary Table 8**. All participants contributed sufficient usable trials under each condition after prespecified cleaning, and no participant-level exclusions were required. For outcomes with effective n < 12, this reflects zero paired differences rather than missing data or participant exclusions. Reaction times decreased from camp entry to camp exit for both game-related words (exact p=0.016) and neutral words (exact p=0.042). Accuracy increased in both conditions, with higher post-camp accuracy for game-related words (exact p=0.010) and neutral words (exact p=0.002). Changes in interference indices did not reach statistical significance (**Table 4**). Accordingly, Stroop findings were interpreted as exploratory changes in overall performance rather than as direct evidence that the camp produced a specific short-term change in interference control. Individual-level trajectories were directionally consistent with faster responses and higher accuracy at camp exit.

**Table 4:** Stroop task performance at camp entry and camp exit (n=12).

Outcome	Camp entry (Pre)	Camp exit (Post)	Δ (Post−Pre)	HL median diff	95% CI (HL)	Wilcoxon exact p (two-sided)	Effect size r	Cohen’s dz
RT (game-related), ms	1030.41 ± 235.89; 987.77 (906.67, 1125.83) [691.13, 1638.10]	877.92 ± 303.96; 846.20 (614.43, 1014.70) [546.62, 1561.38]	-152.49 ± 188.35 [-476.12, 72.91]	-151.05	-276.42 to -18.86	0.016	0.679	0.810
RT (neutral), ms	1033.25 ± 244.49; 1013.67 (854.33, 1112.34) [702.45, 1612.15]	888.83 ± 311.17; 842.67 (639.89, 1045.09) [534.59, 1614.40]	-144.41 ± 180.81 [-416.48, 89.56]	-148.45	-277.42 to -0.04	0.042	0.589	0.799
Accuracy (game-related)	0.870 ± 0.100; 0.880 (0.860, 0.940) [0.620, 0.960]	0.930 ± 0.060; 0.950 (0.930, 0.960) [0.760, 0.980]	0.060 ± 0.060 [-0.050, 0.170]	0.060	0.020 to 0.100	0.010	0.751	0.883
Accuracy (neutral)	0.880 ± 0.080; 0.910 (0.800, 0.940) [0.770, 0.980]	0.950 ± 0.040; 0.960 (0.910, 0.960) [0.880, 1.000]	0.070 ± 0.060 [-0.020, 0.180]	0.060	0.020 to 0.100	0.002	0.815	1.092
RT interference index	-2.84 ± 76.17; -2.21 (-33.65, 44.07) [-171.73, 114.82]	-10.91 ± 41.14; -7.84 (-53.03, 21.17) [-70.11, 60.14]	-8.07 ± 69.46 [-90.53, 161.77]	-13.97	-52.96 to 35.62	0.470	0.226	0.116
Accuracy interference index	-0.010 ± 0.060; -0.020 (-0.020, 0.010) [-0.160, 0.070]	-0.020 ± 0.040; -0.010 (-0.030, 0.000) [-0.120, 0.020]	-0.010 ± 0.060 [-0.140, 0.050]	0.000	-0.050 to 0.030	0.910	0.045	0.178

Δ=Post−Pre. Interference indices were computed as game-related minus neutral (RT interference index=RT_game−RT_neutral; accuracy interference index=Acc_game−Acc_neutral). HL denotes the Hodges–Lehmann estimate of the median paired difference. 95% CIs were obtained using the distribution-based method associated with the Wilcoxon signed-rank procedure. Wilcoxon signed-rank tests are two-sided; exact p values are reported in this table. Effect size r=|Z|/√n_e_ff, where effective n is the number of non-zero paired differences. Cohen’s d^z^=|mean(Δ)|/SD_Δ. Standardized Z and asymptotic p values are provided in **Supplementary Table S8**.

### 3.6 Process measures and acceptability

**Figure 4:**
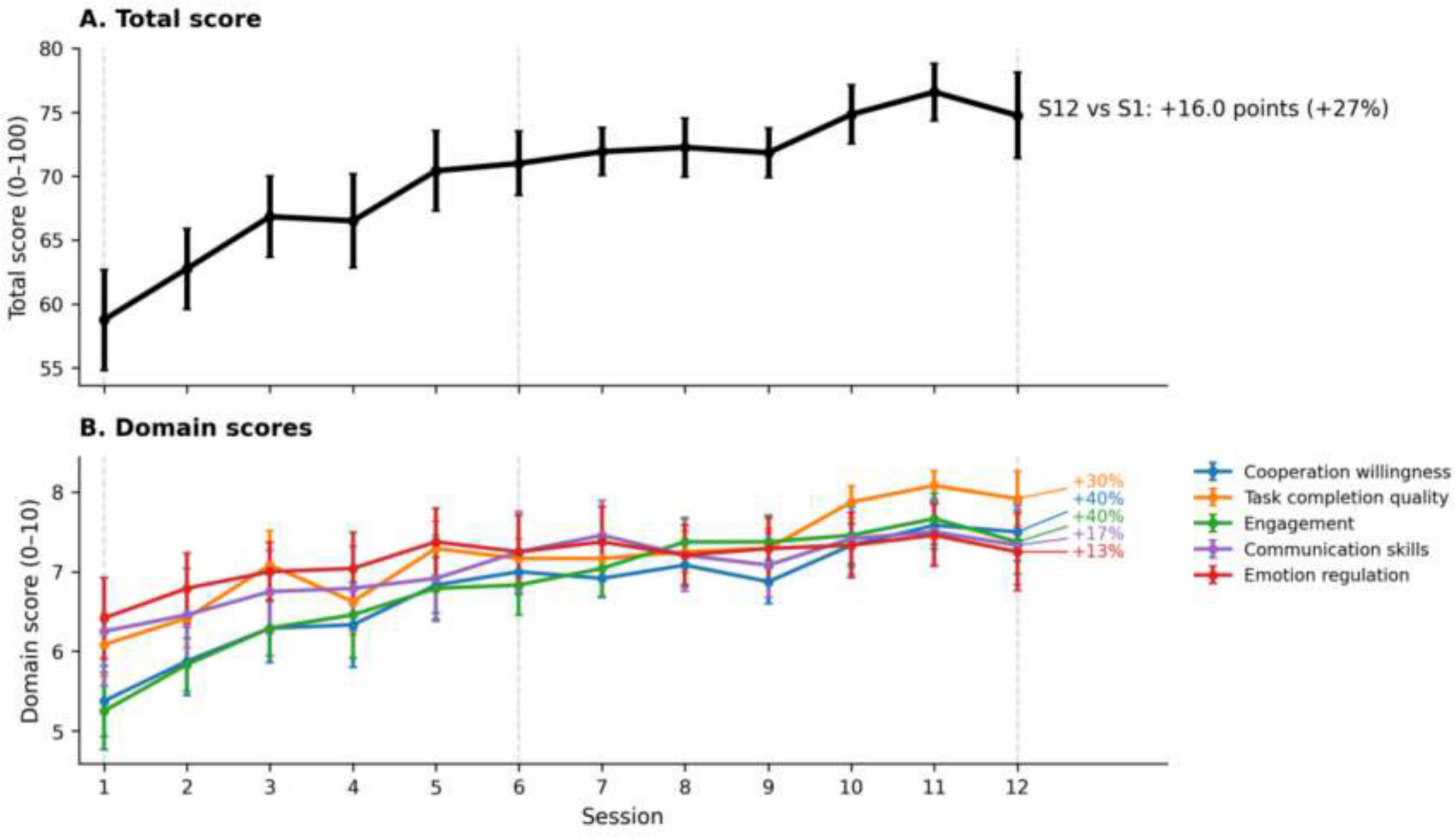
Counselor observational ratings across 12 sessions (n=12). Values are mean ± SE across participants to visualize uncertainty around the mean. Domain scores range from 0 to 10 and the total score ranges from 0 to 100 according to the prespecified scoring rules; corresponding mean ± SD values are reported in **Table 5** and **Supplementary Table 9**.

Session-level counselor observation ratings, scored as five domain means (0–10 each) and one total score based on the sum of 10 subitems (0–100), suggested progressive increases across the 12 sessions (**Figure 4**; **Table 5**; **Supplementary Table 9**), with the mean total score increasing from 58.8 at Session 1 to 74.8 at Session 12 (+16.0 points, +27%). Post-camp acceptability was generally favorable but heterogeneous (**Supplementary Figure 3**; **Supplementary Table 10**), with a single-item overall experience rating of 4.17 ± 1.11 (1–5 scale) and the highest item-level ratings for staff support (4.33 ± 0.89) and organization/management (4.25 ± 0.97).

**Table 5:** Key time-point comparisons of counselor observational ratings (n=12).

Domain	S1 (mean ± SD)	S6 (mean ± SD)	S12 (mean ± SD)	Change vs S1 (%)
Cooperation willingness	5.38 ± 1.54	7.00 ± 0.98	7.50 ± 1.26	+40%
Task completion quality	6.08 ± 1.36	7.17 ± 0.86	7.92 ± 1.18	+30%
Engagement	5.25 ± 1.69	6.83 ± 1.30	7.38 ± 1.40	+40%
Communication skills	6.25 ± 2.35	7.25 ± 1.75	7.33 ± 1.72	+17%
Emotion regulation	6.42 ± 1.77	7.25 ± 1.60	7.25 ± 1.70	+13%
Total score (0-100)	58.75 ± 13.65	71.00 ± 8.69	74.75 ± 11.66	+27%

Key time points were defined as Session 1 (start), Session 6 (mid-camp), and Session 12 (end). Five domains were rated after each session. Each domain score ranges from 0 to 10 and was calculated as the mean of two subitems scored 0–10; the total score ranges from 0 to 100 and was calculated as the sum of all 10 subitems. Values are mean ± SD.

### 3.7 Safety and adverse events

No serious adverse events occurred during the camp, and no participant discontinued. Safety events and their management are summarized in **Supplementary Table 3**.

### 3.8 Exploratory association analyses

Exploratory Spearman correlations between changes in the primary outcomes and changes in other measures are summarized in **Supplementary Table 11**. A broader, descriptive correlation heatmap across all available change scores is shown in **Supplementary Figure 4**. Changes in GDSS and GAS-7 were strongly correlated (ρ=0.750, p=0.005), consistent with concordant change across primary IGD severity measures. Greater reductions in craving were associated with larger increases in IRI-C total scores/self-reported interpersonal responsiveness (ΔVAS vs ΔIRI-C: ρ=−0.675, p=0.016). Baseline PSQI global score was positively correlated with ΔVAS (ρ=0.767, p=0.004), suggesting smaller decreases in craving among adolescents with poorer baseline sleep quality; because PSQI was a baseline-only measure, this finding should be interpreted as an exploratory baseline-to-change association rather than as evidence of change in sleep during the camp. All other associations between primary outcome changes and secondary/process measures were weak and did not reach nominal significance, and findings were interpreted cautiously given the exploratory design and lack of multiple-comparison correction.

## 4 Discussion

In this retrospective, de-identified, single-group program evaluation, we examined the feasibility, safety, and short-term entry-to-exit signals of a 7-day structured residential camp in 12 adolescents aged 11–16 years with DSM-5 Internet Gaming Disorder. The main contribution is not causal efficacy evidence, but documentation of an underreported service format: a brief, context-controlled residential program delivered in routine care with feasibility, safety, process, and multi-informant outcome data. The program used face-to-face, rule-governed games to deliver cognitive–behavioral and motivational elements while temporarily reducing high-salience gaming cues. Existing intervention evidence remains heterogeneous, and intensive camp-based models remain under-evaluated (8, 12). Accordingly, all observed outcome changes should be interpreted as preliminary and hypothesis-generating rather than definitive.

Camp-exit outcomes showed a concordant directional signal across the three core measures: GDSS decreased in all 12 adolescents, GAS-7 decreased in 11 with 1 unchanged, and VAS craving decreased overall with meaningful inter-individual variability. The small increase in craving observed in one participant may reflect individual heterogeneity, exit-related anticipation, or emotional activation during the camp, but no participant-level qualitative data were available to determine the cause. Reductions in severity and craving are clinically interpretable in this pilot context because craving is proximal to cue reactivity and reinforcement processes (30, 31). Prespecified threshold-based indices suggested a descriptive reduction in high-risk status and a preliminary responder signal, broadly consistent with prior camp and structured short-term treatment work (12, 32). However, the residential group setting itself may also have contributed through peer modeling, group cohesion, shared routines, social reinforcement, and counselor-supported group norms. Therefore, the present single-group design cannot separate contextual cue reduction, group dynamics, and game-embedded learning processes.

The secondary-domain signals should be interpreted as compatible with, rather than proof of, the hypothesized change model. Team games may have operationalized rules, feedback, cooperative tasks, and structured debriefing to rehearse persistence, offline social reward, emotion regulation, and craving coping. However, the study did not include direct mechanistic measures or a dismantling design; therefore, changes in impulsivity, counselor-rated observable behavioral dysregulation, and self-reported interpersonal responsiveness cannot be attributed to any specific active ingredient.

Change was not uniform over the 7-day window. Anxiety and social avoidance showed no clear statistical signal, which is plausible because these domains may be bidirectionally linked with gaming or reflect longer-standing vulnerability requiring longer transfer windows (33, 34). In contrast, depressive symptoms, IRI-C total scores, and counselor-rated behavioral dysregulation showed more favorable short-term shifts, but these should remain supportive or exploratory signals rather than evidence of broad psychosocial recovery. Similarly, Stroop performance showed faster reaction time and higher accuracy without significant change in interference indices. Because reaction time and accuracy can reflect familiarity, strategy, motivation, and test–retest limitations, these findings should not be interpreted as evidence of robust short-term improvement in inhibitory control (35, 36).

Process and acceptability data are important because this was a complex, multi-component residential program rather than a single manualized technique (37, 38). Completion, attendance, session observations, and satisfaction ratings supported feasibility and engagement, while the combination of self-report, counselor ratings, and in-camp observations reduced reliance on a single informant (39). Camp residential counselor-rated Conners scores were useful as a structured, non-self-report observation of behavioral dysregulation across repeated rule-governed tasks and daily routines, where attention, impulsivity/hyperactivity, task persistence, and behavioral regulation could be observed beyond self-report questionnaires. These ratings remained context-bound exploratory observations and may be sensitive to rater expectations and the structured camp environment; more broadly, because counselor ratings were unblinded and self-reports may be affected by social desirability and context, subjective findings should be interpreted within bias boundaries (40). Exploratory correlations also provided hypothesis-generating context: poorer baseline sleep quality was associated with smaller craving reduction, and craving reduction covaried with increases in IRI-C total scores/self-reported interpersonal responsiveness. These findings are consistent with prior evidence linking sleep problems to IGD-related behavioral dysregulation and adolescent risk trajectories (41, 42), but should not be interpreted as mechanistic evidence given the small sample, baseline-only PSQI assessment, and lack of multiplicity correction.

Findings should be interpreted within the constraints of a retrospective, small, single-group entry-to-exit evaluation. The referral and screening pool was not prospectively recorded; the sample was developmentally heterogeneous; concomitant treatments and medication details were not systematically extracted; baseline gaming exposure may have been affected by pre-entry restrictions; several self-report instruments had recall windows longer than the 7-day interval; and no follow-up was available to assess durability. Baseline-only PSQI and FACES II-CV could only provide contextual information, and subjective or counselor-rated outcomes remained vulnerable to expectancy and context effects. The Stroop task was exploratory and may have been affected by retest or practice influences. The design also cannot disentangle environmental restriction, group dynamics, counselor-supported routines, and game-embedded therapeutic mechanisms, a limitation shared by prior camp work (12). Future studies should use controlled designs with follow-up, objective gaming/screen metrics, developmentally tailored or age-stratified delivery, and matched-context or dismantling comparisons. Transparent reporting should continue to integrate implementation and outcome evidence using complex-intervention and pilot-trial guidance (37, 38); therefore, these findings should be read as early implementation and short-term signal data rather than stand-alone evidence of treatment efficacy.

## 5 Conclusion

In this retrospective single-group entry-to-exit pilot evaluation, a 7-day closed, structured gaming-based camp program was feasible and safe, with complete retention and high attendance. The observed entry-to-exit reductions in core Internet gaming disorder severity and craving should be interpreted as short-term preliminary signals rather than definitive evidence of treatment efficacy. The main value of the present study is to provide implementation-oriented evidence for a brief, highly structured service format and to inform future controlled studies with longer follow-up, including work to disentangle contextual cue restriction from game-embedded therapeutic ingredients, to identify clinically relevant moderators such as baseline sleep problems, and to clarify whether developmentally tailored or age-stratified delivery is needed across mixed adolescent samples.

## Data Availability

The original contributions presented in the study are included in the article/**Supplementary Material**. Further inquiries can be directed to the corresponding author.
