# Supplementary material for: Gaming-based program for internet gaming disorder: feasibility and preliminary outcomes of a structured camp program
Source: Front Psychiatry. 2026 Jun 9;17:1825298. doi: 10.3389/fpsyt.2026.1825298 (PMC13288209; doi:10.3389/fpsyt.2026.1825298)
Supplement: Supplementary file 1 [file Supplementaryfile1.docx]

**Supplementary Figure S1. Participant flow for the enrolled sample (screening pool size not systematically recorded).**


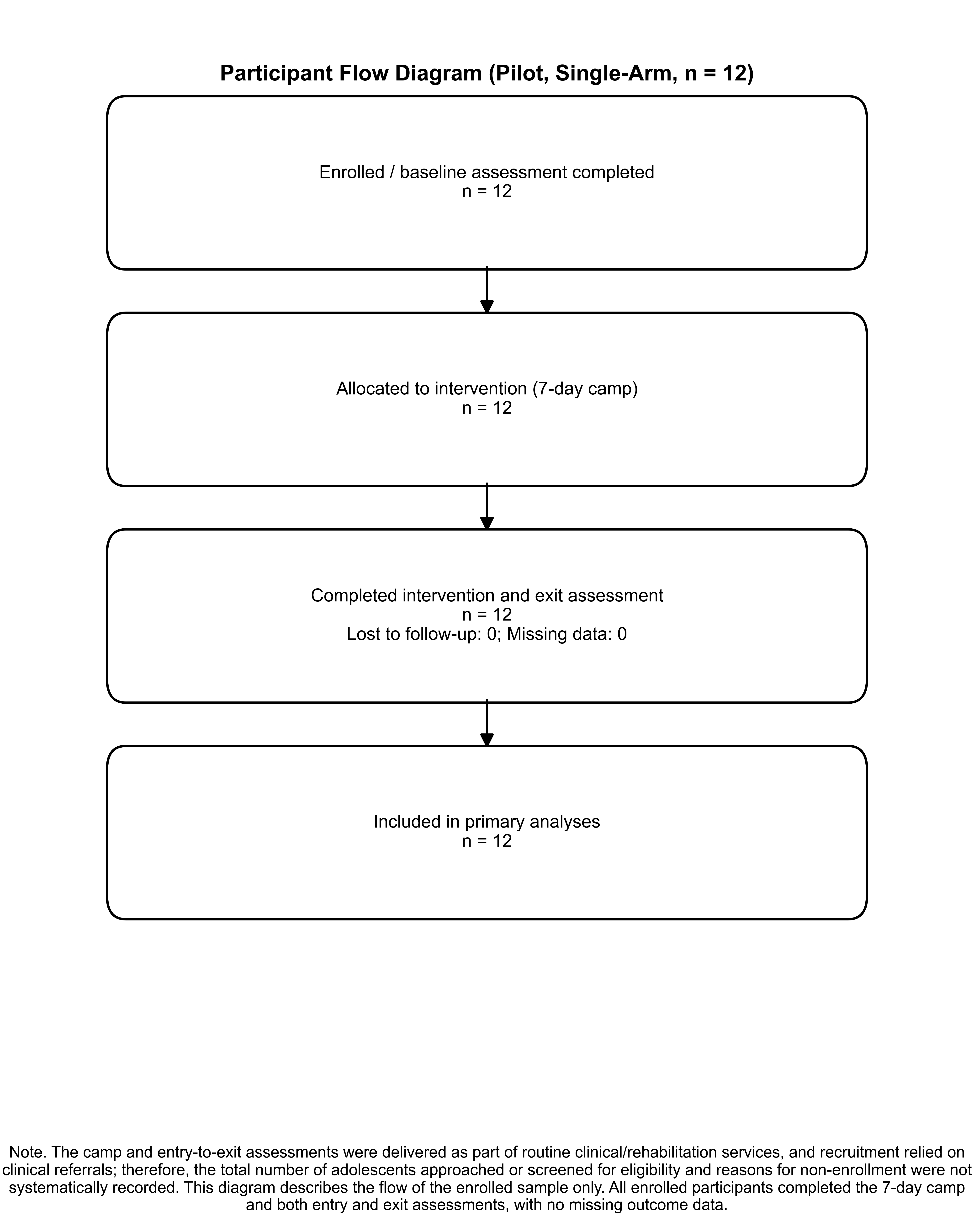


*Note. The camp and entry-to-exit assessments were delivered as part of routine clinical/rehabilitation services, and recruitment relied on clinical referrals; therefore, the total number of adolescents approached or screened for eligibility and reasons for non-enrollment were not systematically recorded. This diagram describes the flow of the enrolled sample only. All enrolled participants completed the 7-day camp and both entry and exit assessments, with no missing outcome data.*

**Supplementary Figure S2. Prespecified threshold-based rates (Pre vs Post)**

***
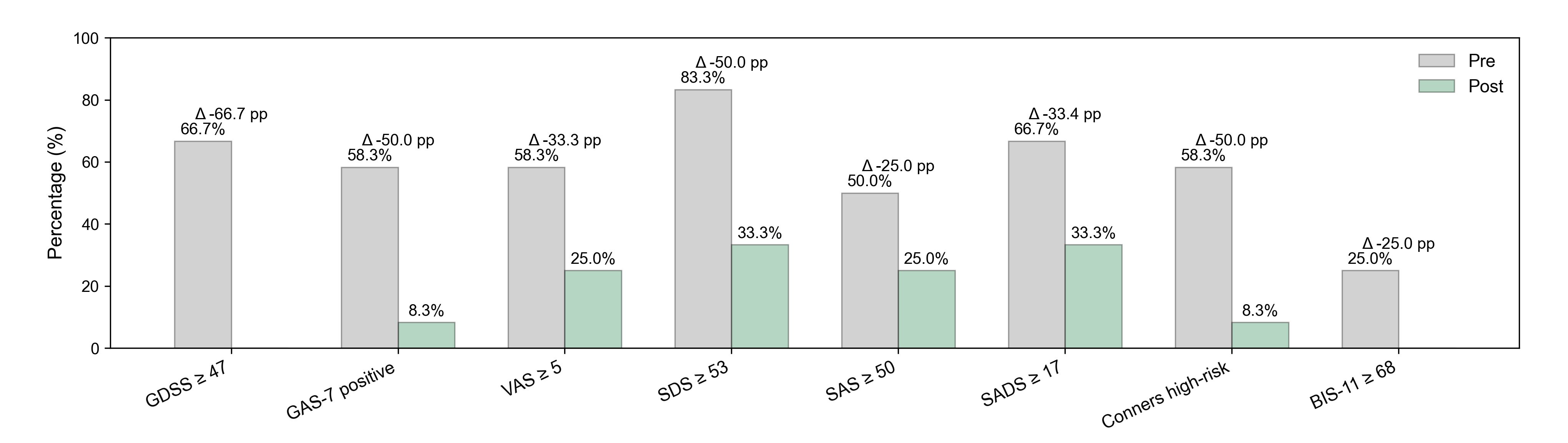
***

*Notes. Rates indicate the proportion meeting each prespecified threshold (n=12). Threshold definitions are provided in Supplementary Table S6. GAS-7 positivity was defined using the polythetic rule (≥4 of 7 items scored ≥3). Conners “high-risk” was defined as Hyperactivity Index mean ≥1.5. Conners thresholds are reported descriptively only because ratings were completed by camp residential counselors as exploratory structured observations in the residential context, not as diagnostic ADHD screening or standard school-based teacher ratings. SADS thresholds are reference cutoffs only. Δ denotes the change in percentage points (Post−Pre); percentages are rounded and Δ may differ slightly due to rounding. Bars with 0% at a time point may have zero height and appear visually absent.*

**Supplementary Figure S3. Participant satisfaction ratings with camp activities and logistics**

**
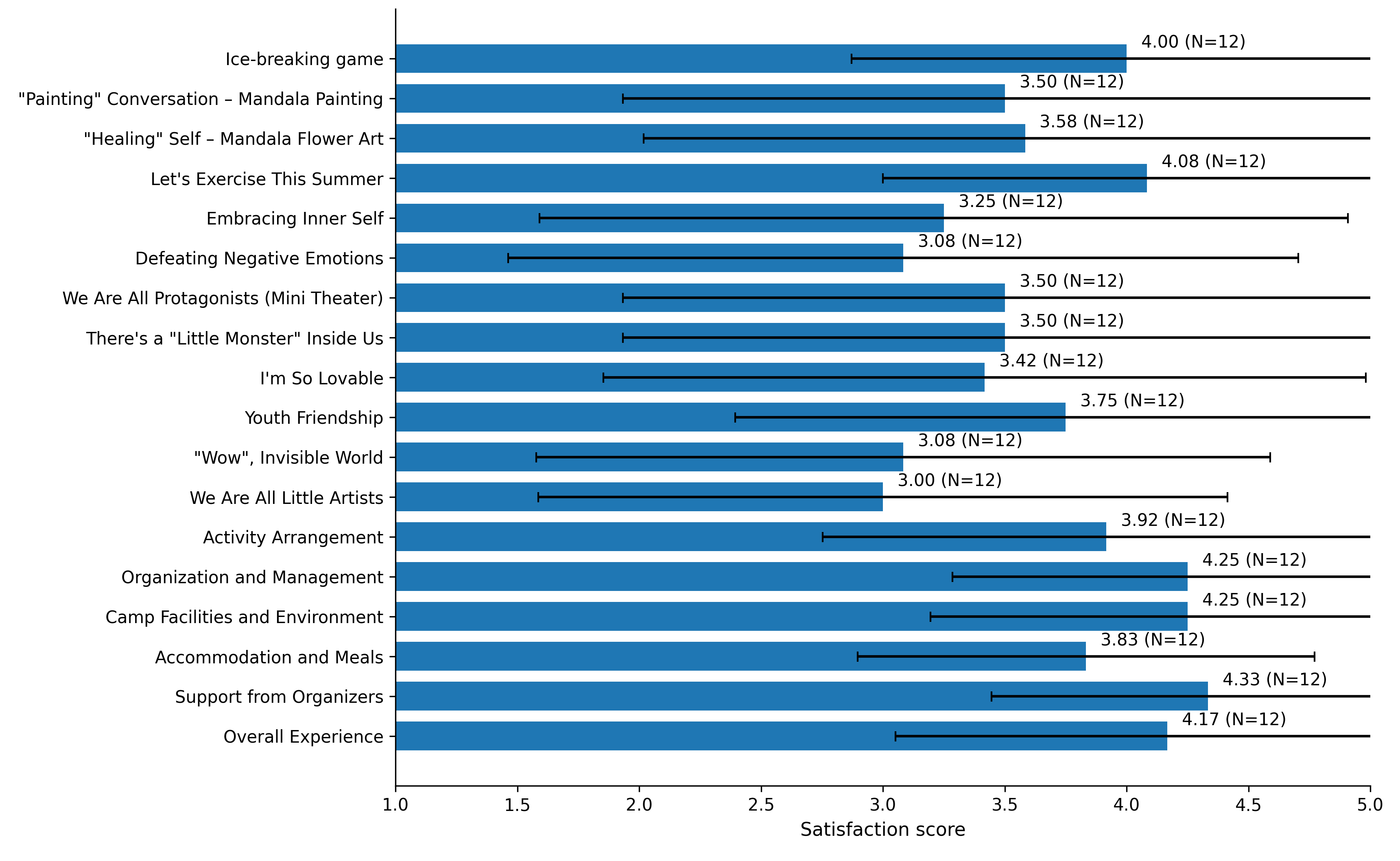
**

*Notes. Ratings were collected at camp exit. Bars show mean satisfaction ratings (1–5) and error bars indicate standard deviation. Higher scores indicate greater satisfaction.*

**Supplementary Figure S4. Spearman correlation heatmap of change scores (Δ=Post−Pre) (exploratory, n=12)**

**
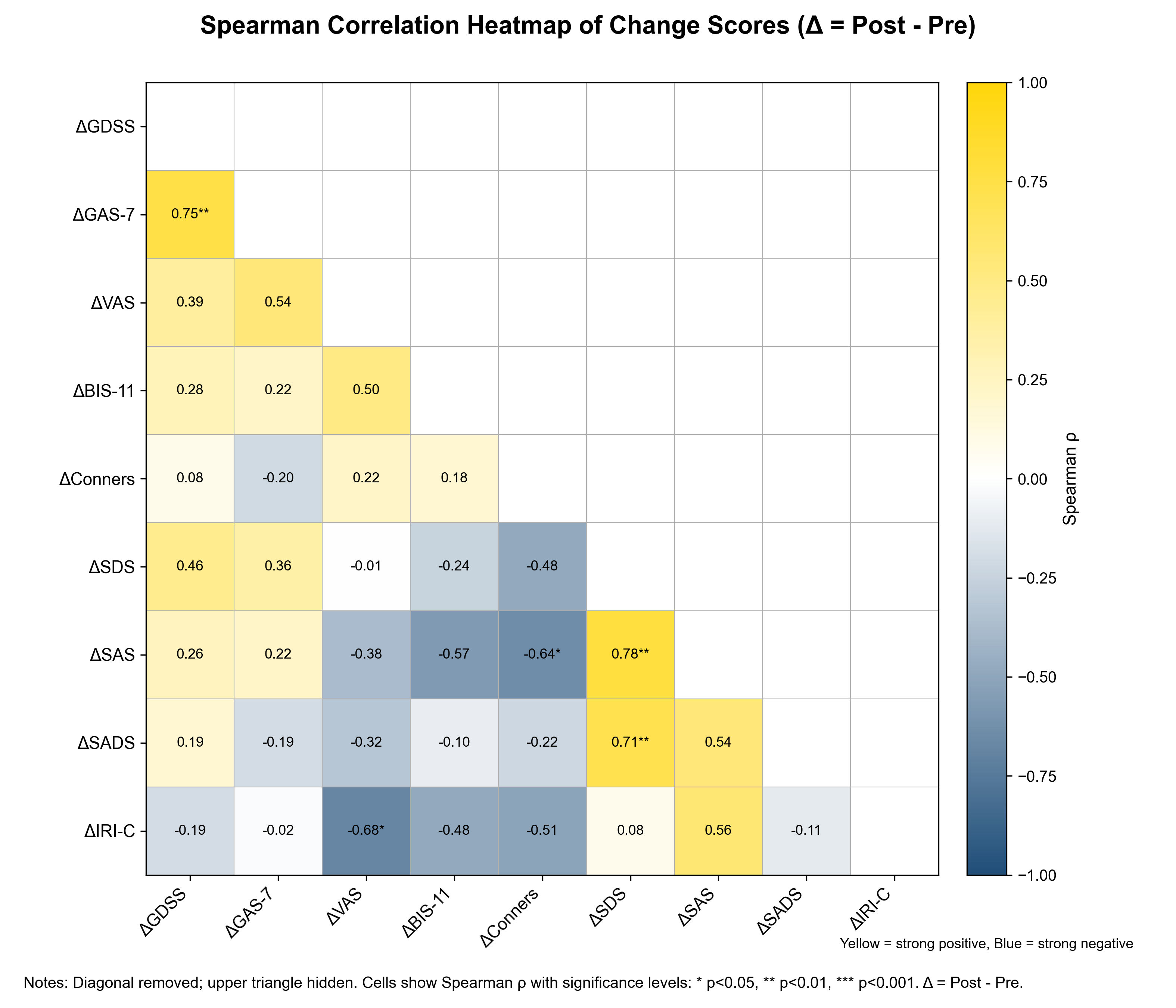
**

*Notes. The diagonal is omitted and the upper triangle is hidden. Cells display Spearman’s ρ based on change scores (Δ=Post−Pre). For symptom measures (GDSS, GAS-7, VAS, BIS-11, Conners, SDS, SAS, SADS), more negative Δ indicates larger decreases; for IRI-C, more positive Δ indicates larger increases in self-reported interpersonal responsiveness. Asterisks indicate nominal significance (* p<0.05; ** p<0.01; *** p<0.001). No multiple-comparison correction was applied; results are exploratory.*

**Supplementary Table S1. Intervention dose and components of the 7-day game-based therapeutic camp for Internet gaming disorder (n=12).**

| **Session** | **Session title** | **Therapeutic target (IGD)** | **Gamification mechanisms and key techniques** | **Format** | **Residential counselor role (participant + observer)** | **Homework (as assigned)** |
| --- | --- | --- | --- | --- | --- | --- |
| 1 | Ice-breaking game | Build therapeutic alliance and group rules; initiate alternative reinforcement in real-world contexts. | Cooperative onboarding challenges with team points and rule-based unlocking; emphasis on rule adherence and immediate feedback to re-anchor reward contingencies. | Group game | Co-participate to maintain rules and order; observe and rate Domains A–E after the session. | None |
| 2 | "Painting" Conversation – Mandala Painting | Reduce hyperarousal and impulsive responding; train sustained attention and delay of gratification. | Progression-based tasks with stepwise feedback and “clues”; structured advancement replaces instant-scroll reinforcement. | Group + individual | Support task boundaries; document persistence, distractibility, and affective fluctuations; post-session ratings. | None |
| 3 | "Healing" Self – Mandala Flower Art | Enhance self-efficacy and non-gaming achievement experiences. | Achievement-leveling plus structured peer feedback strengthens effort → output → recognition loop. | Group | Facilitate expression and feedback; record engagement and communication; post-session ratings. | Daily achievement log |
| 4 | Let's Exercise This Summer | Provide alternative reinforcement and regulate physiological arousal to reduce craving-driven behavior. | Team-based activity challenges with bonus points shift high stimulation toward physical activity and cooperation. | Group exercise | Accompany participation; ensure safety and order; post-session ratings. | None |
| 5 | Embracing Inner Self | Support identity reconstruction and self-acceptance; reduce reliance on gaming identity. | Narrative gamification using character cards and skill trees translating real-life strengths into “upgradeable skills.” | Group | Support disclosure; prevent withdrawal; record communication and emotion; post-session ratings. | None |
| 6 | Defeating Negative Emotions | Train emotion identification and regulation; reduce emotion-avoidant gaming. | “Boss battle” metaphor: trigger mapping, coping skills as “abilities,” and debrief as replay; strengthens alternative coping. | Group | Monitor escalation; apply de-escalation when needed; record task completion and emotion regulation; ratings. | None |
| 7 | We Are All Protagonists (Mini Theater) | Rehearse social skills and roles to increase real-world social reinforcement. | Scenario-based role-play levels: initiating conversation, expressing needs, resolving conflicts; immediate feedback and debrief. | Group performance | Organize roles and rules; record communication and engagement; post-session ratings. | Write one improved line for next time |
| 8 | There's a "Little Monster" Inside Us | Externalize craving and impulses to increase psychological distance and self-control. | Quest-chain framing: labeling craving as an external “monster,” practicing pause-and-choose responses and alternative actions. | Group | Observe impulsive moments; support interrupting impulse chains; post-session ratings. | None |
| 9 | I'm So Lovable | Build self-esteem and acceptance; reduce compensatory gaming. | Cooperative tasks with structured positive feedback; “assist/like” mechanism strengthens interpersonal reward. | Group | Facilitate inclusion and positive interaction; record cooperation and communication; ratings. | Write three examples of real-life recognition |
| 10 | Youth Friendship | Enhance peer relationships and belonging; reduce social avoidance. | Team quest cards: asking for help, expressing gratitude, proactive joining; cooperative accomplishment over win–lose competition. | Group | Bridge interactions; document avoidance and participation; ratings. | None |
| 11 | "Wow", Invisible World | Rebuild attentional shifting and curiosity; replace screen stimulation with real-world exploration. | Treasure-hunt design: clue system + task-driven exploration; reinforces immersion and satisfaction without screens. | Group | Ensure safety; participate throughout; record engagement and emotion; ratings. | None |
| 12 | We Are All Little Artists | Consolidation and relapse prevention; develop a maintenance plan. | Final quest + “save system”: review gains, generate coping scripts for high-risk situations, and complete a maintenance plan. | Group wrap-up | Support synthesis and emotion processing; record communication; post-session ratings. | Maintenance plan worksheet |

*Note. This 7-day camp program comprised 12 structured sessions within an integrated psychosocial framework targeting key mechanisms of Internet gaming disorder. It combined cognitive–behavioral and motivational elements with emotion-regulation practice, alternative offline reinforcement, social-skills rehearsal, identity and self-efficacy work, and relapse-prevention planning, while gamification strategies were used to structure engagement, feedback, and graded task participation. Residential counselors accompanied participants throughout all contexts (sessions, meals, accommodation), serving as both participants to support rule enforcement and teamwork and as observers to provide structured behavioral assessment and immediate feedback. Participants were not permitted to bring personal mobile phones or internet-enabled devices to the camp; throughout the camp they had no access to smartphones and were fully separated from online gaming. Parents were informed and agreed, and necessary family contact was arranged once nightly via supervised voice calls using counselors’ phones. For participants requiring medication, hospital nurses prepared and dispensed daily medications, and residential counselors supervised each dose to ensure adherence throughout the camp. At camp exit, participants retrospectively rated satisfaction for each of the 12 sessions and six program logistics items on a 1–5 Likert scale (1=very dissatisfied, 5=very satisfied); overall satisfaction was computed as the mean across all 18 items. After each session, counselors rated performance in five domains: cooperation willingness, task completion quality, engagement, communication ability, and emotion regulation. Each domain score ranged from 0 to 10 and was calculated as the mean of two subitems scored 0–10; the total score ranged from 0 to 100 and was calculated as the sum of all 10 subitems. Higher scores indicate better performance.*

**Supplementary Table S2. Feasibility and fidelity.**

| **Metric** | **Value** | **Definition / recording** |
| --- | --- | --- |
| Enrolled participants | 12 | Single-arm pilot study. |
| Completion rate | 12/12 (100%) | Completed the 7-day program and the camp-exit assessment. |
| Overall program delivery | All sessions delivered as scheduled | No session cancellations or overall shortening. |
| Attendance rate (completed participation) | 142/144 (98.6%) | Attendance based on completed participation: 142/144 session-person counts; 10 participants at 100% and 2 at 91.7%. |
| Brief session interruption / partial absence | 2/144 session-person counts (1.4%) | Two participants had a brief step-out during the ‘Defeating Negative Emotions’ session due to marked emotional reactions; per the prespecified attendance rule, this session-person was coded as non-attendance because the full session was not completed, although both participants returned and participated in the remainder of the session. No further interruptions occurred, and both participants completed the camp-exit assessment. |
| Subsequent participation and assessment completion | Yes (2/2) | The brief interruption occurred only during that single session. |
| Group conflict incident | 1 incident | One interpersonal conflict occurred on day 3 (two participants involved); de-escalated after on-site psychiatric mediation; both completed subsequent sessions. |
| Protocol deviations | None | Temporary pauses or additional emotional support were part of safety management and did not change the core intervention content. |
| Data completeness | Missing questionnaires: 0 | No missing post-intervention questionnaires for pre–post analyses; PSQI and FACES II-CV were baseline-only measures. |

*Note. Attendance was calculated as completed participation using session-person counts, defined as the number of participants multiplied by the number of planned sessions (12×12=144). Brief session interruption or partial absence was defined as a temporary step-out from a session due to acute emotional escalation requiring supportive management, followed by return and completion of the remainder of the session; this was not treated as withdrawal from the camp program. “Group conflict” was recorded as an incident-level event; the number of involved participants is reported in Supplementary Table S3. Per the prespecified attendance coding rule, any partial absence during a session was coded as non-attendance for that session, contributing two missed session-counts (2/144).*

**Supplementary Table S3. Safety and adverse events.**

| **Event** | **n (%)** | **Description** | **Severity** | **Relatedness** | **Management and outcome** |
| --- | --- | --- | --- | --- | --- |
| Marked emotional escalation (brief session interruption) | 2 (16.7%) | During “Defeating negative emotions”, one male and one female participant had marked emotional reactions requiring a brief time-out; both returned and completed the remainder of the session. | Mild–moderate | Possibly related | On-site supportive intervention and reassurance were provided. Both participants attended all remaining sessions and completed the camp-exit assessment; per the prespecified attendance coding rule, this interruption was coded as non-attendance for that session-person. |
| Peer conflict/escalation | 2 (16.7%) | On day 3, two male participants had an interpersonal conflict. | Mild–moderate | Possibly related | De-escalated after on-site psychiatric mediation; both completed subsequent sessions and relationship was repaired. |
| Serious adverse events | 0 (0) | No self-harm, no severe aggression, no emergency care required, and no withdrawals. | — | — | — |

*Note. n (%) is based on participants (N=12). Severity was categorized as mild–moderate versus serious. Serious adverse events were defined as events requiring emergency treatment or hospitalization, self-harm/suicidal behavior, or events leading to discontinuation from the camp program. “—” indicates not applicable.*

**Supplementary Table S4. Expanded baseline psychosocial profiles.**

| **Domain** | **Measure** | **Value** | **Notes** |
| --- | --- | --- | --- |
| Sleep quality (PSQI) | PSQI global score, mean ± SD | 8.75 ± 3.72 | Overall sleep quality was poor relative to the standard cutoff of >5 for poor sleep quality. |
| Sleep quality (PSQI) | PSQI global score, median (IQR) | 9.0 (5.0–10.5) |  |
| Sleep quality (PSQI) | PSQI global score, range | 4–16 |  |
| Sleep quality (PSQI) | PSQI >5 (poor sleeper) | 8/12 (66.7%) | Most adolescents met criteria for poor sleep quality. |
| Sleep quality (PSQI) | PSQI >8 (moderate-to-severe sleep problems) | 7/12 (58.3%) | More than half met criteria for moderate-to-severe sleep problems. |
| Sleep quality (PSQI) | Component: Use of sleep medication, mean ± SD | 2.00 ± 1.48 | Rank 1 (highest) |
| Sleep quality (PSQI) | Component: Subjective sleep quality, mean ± SD | 1.50 ± 1.00 | Tied Rank 2 |
| Sleep quality (PSQI) | Component: Sleep latency, mean ± SD | 1.50 ± 1.24 | Tied Rank 2 |
| Sleep quality (PSQI) | Component: Daytime dysfunction, mean ± SD | 1.50 ± 1.09 | Tied Rank 2 |
| Sleep quality (PSQI) | Component: Sleep disturbances, mean ± SD | 1.00 ± 0.85 | Rank 5 |
| Sleep quality (PSQI) | Component: Habitual sleep efficiency, mean ± SD | 0.75 ± 1.06 | Rank 6 |
| Sleep quality (PSQI) | Component: Sleep duration, mean ± SD | 0.50 ± 0.67 | Rank 7 (lowest) |
| Family functioning (FACES II-CV) | Cohesion (16 items) (Actual), mean ± SD | 45.75 ± 6.43 |  |
| Family functioning (FACES II-CV) | Cohesion (16 items) (Ideal), mean ± SD | 59.75 ± 11.44 |  |
| Family functioning (FACES II-CV) | Cohesion (16 items) (Actual − Ideal), mean ± SD | -14.00 ± 12.11 | Negative values indicate perceived functioning below the ideal. |
| Family functioning (FACES II-CV) | Adaptability (14 items) (Actual), mean ± SD | 37.67 ± 9.35 |  |
| Family functioning (FACES II-CV) | Adaptability (14 items) (Ideal), mean ± SD | 54.83 ± 10.29 |  |
| Family functioning (FACES II-CV) | Adaptability (14 items) (Actual − Ideal), mean ± SD | -17.17 ± 13.20 | Negative values indicate perceived functioning below the ideal. |
| Family functioning (FACES II-CV) | Total score (Actual), mean ± SD | 83.42 ± 14.70 |  |
| Family functioning (FACES II-CV) | Total score (Ideal), mean ± SD | 114.58 ± 20.77 |  |
| Family functioning (FACES II-CV) | Total score (Actual − Ideal), mean ± SD | -31.17 ± 22.58 | Negative values indicate perceived functioning below the ideal. |

*Note. Values are presented as mean ± SD, median (IQR), range, or n/N (%), as appropriate. PSQI indicates Pittsburgh Sleep Quality Index; higher scores indicate poorer sleep quality. PSQI was assessed at baseline only and is reported to characterize baseline sleep-impairment burden rather than short-term treatment response; both PSQI >5 and PSQI >8 are presented for descriptive characterization of sample severity. FACES II-CV indicates Family Adaptability and Cohesion Evaluation Scales II, Chinese Version and is reported as a baseline family-context indicator only. Gap score is defined as Actual − Ideal, where negative values indicate perceived actual functioning below the ideal.*

**Supplementary Table S5. Wilcoxon Z and asymptotic p values for primary outcomes**

| **Outcome** | **nₑff** | **Z** | **asymptotic p (two-sided)** |
| --- | --- | --- | --- |
| GDSS total | 12 | -3.066 | 0.002 |
| GAS-7 total | 11 | -2.952 | 0.003 |
| VAS craving (0–10) | 9 | -2.556 | 0.011 |

*Notes. Effective n denotes the number of non-zero paired differences. Z was computed using the normal approximation to the Wilcoxon signed-rank test with tie correction based on absolute paired differences, and asymptotic p is the two-sided asymptotic p value derived from Z. Δ is defined as camp-exit minus camp-entry. Exact p values are reported in the main manuscript (Table 2).*

**Supplementary Table S6. Threshold-based responder and descriptive clinical stratification analyses.**

| **Measure** | **Prespecified threshold or stratification rule** | **Pre positive, n/N (%)** | **Post positive, n/N (%)** | **Positive→negative, n** | **Negative→positive, n** | **Responder definition** | **Responder outcome** |
| --- | --- | --- | --- | --- | --- | --- | --- |
| GDSS | High risk: total score ≥47; severe: total score ≥54 | ≥47: 8/12 (66.7%); ≥54: 5/12 (41.7%) | ≥47: 0/12 (0.0%); ≥54: 0/12 (0.0%) | ≥47: 8; ≥54: 5 | ≥47: 0; ≥54: 0 | ≥50% reduction in total score | 9/12 (75.0%) |
| GAS-7 (polythetic scoring, primary) | Positive: ≥4 of 7 items scored ≥3 | 7/12 (58.3%) | 1/12 (8.3%) | 6 | 0 | Positive-to-negative | 6/12 (50.0%) |
| GAS-7 (monothetic scoring, sensitivity) | Positive: all 7 items scored ≥3 | 2/12 (16.7%) | 0/12 (0.0%) | 2 | 0 | Positive-to-negative | 2/12 (16.7%) |
| VAS craving | Moderate-to-severe craving: ≥5; low craving: ≤3; no craving: 0 (reported separately as a subset of ≤3) | ≥5: 7/12 (58.3%); ≤3: 4/12 (33.3%); =0: 2/12 (16.7%) | ≥5: 3/12 (25.0%); ≤3: 8/12 (66.7%); =0: 3/12 (25.0%) | ≥5: 4 | ≥5: 0 | Paired trajectory: decrease / no change / increase | Decrease: 8/12 (66.7%); No change: 3/12 (25.0%); Increase: 1/12 (8.3%) (3→4) |
| SDS (standard score) | Positive: ≥53; severe: ≥73 | ≥53: 10/12 (83.3%); ≥73: 2/12 (16.7%) | ≥53: 4/12 (33.3%); ≥73: 0/12 (0.0%) | ≥53: 6; ≥73 remitted: 2 | ≥53: 0 | Positive-to-negative | 6/12 (50.0%) |
| SAS (standard score) | Positive: ≥50; moderate-to-severe: ≥60 | ≥50: 6/12 (50.0%); ≥60: 2/12 (16.7%) | ≥50: 3/12 (25.0%); ≥60: 1/12 (8.3%) | ≥50: 3; ≥60 remitted: 1 | ≥50: 0 | Positive-to-negative | 3/12 (25.0%) |
| BIS-11 (total) | High impulsivity: total score ≥68 | 3/12 (25.0%) | 0/12 (0.0%) | 3 | 0 | From ≥68 to <68 | 3/12 (25.0%) |
| SADS (total) | Reference cutoffs: ≥17 (marked); ≥19 (severe) | ≥17: 8/12 (66.7%); ≥19: 8/12 (66.7%) | ≥17: 4/12 (33.3%); ≥19: 4/12 (33.3%) | ≥17: 4 | ≥17: 0 | Transition out of high-score range | 4/12 (33.3%) |
| Conners Teacher Rating Scale (camp residential counselor–rated) | High ADHD risk: Hyperactivity Index mean ≥1.5; abnormal: any factor mean ≥1.5 | 7/12 (58.3%); 11/12 (91.7%) | 1/12 (8.3%); 2/12 (16.7%) | High-risk: 6; Abnormal: 9 | High-risk: 0; Abnormal: 0 | No formal responder definition; descriptive transition only | Descriptive transition only: high-risk reference 6/12; abnormal reference 9/12 |
| IRI-C | No established clinical cutoff; directional change only | — | — | NA | NA | Directional change (no cutoff) | NA (no clinical cutoff); see pre–post analyses. |

*Note. Values are reported as n/N (%), unless otherwise specified. Pre and Post refer to baseline (camp entry) and post-intervention (camp exit). For GDSS, GAS-7, VAS, BIS-11, SDS, SAS, SADS, and Conners, higher scores indicate greater symptom burden or behavioral dysregulation; For IRI-C, higher total scores were interpreted cautiously as greater self-reported interpersonal responsiveness in this study; no positivity or responder rates were calculated. Positive-to-negative indicates meeting the prespecified cutoff at baseline but not at post-intervention; negative-to-positive indicates the opposite. GDSS high-risk cutoff was total score ≥47 and severe cutoff ≥54; responders were defined as ≥50% reduction in GDSS total score. GAS-7 positivity was defined using polythetic scoring (≥4 of 7 items scored ≥3) as the primary definition; monothetic scoring (all 7 items ≥3) was used as a sensitivity definition. VAS ranges from 0 to 10 with higher scores indicating stronger craving; moderate-to-severe craving was defined as ≥5 and low craving as ≤3; no craving was defined as 0 and is reported separately as a subset of ≤3; paired trajectories are reported as decrease/no change/increase. SDS and SAS use standard scores; positivity thresholds were ≥53 and ≥50, respectively; higher thresholds were reported for descriptive severity stratification. BIS-11 high impulsivity was defined as total score ≥68. SADS cutoffs (≥17 and ≥19) were descriptive reference thresholds only. For Conners, high ADHD risk was defined as Hyperactivity Index mean ≥1.5, and abnormality as any factor mean ≥1.5; both are reference cutoffs only. In this study, Conners ratings were completed by camp residential counselors and were used as exploratory structured observations of behavioral dysregulation in the residential camp context. Thresholds are reported descriptively only and should not be interpreted as diagnostic classification, standard ADHD screening, or equivalent to school-based teacher ratings. IRI-C has no established clinical cutoff; therefore no positivity/responder rates are reported.*

**Supplementary Table S7. Wilcoxon Z and asymptotic p values for secondary outcomes**

| **Outcome** | **nₑff** | **Z** | **asymptotic p (two-sided)** |
| --- | --- | --- | --- |
| BIS-11 total | 12 | -2.946 | 0.003 |
| Conners (camp residential counselor–rated) total | 12 | -3.059 | 0.002 |
| SDS (standard score) | 12 | -2.595 | 0.009 |
| SAS (standard score) | 11 | -1.561 | 0.119 |
| SADS total | 10 | -1.177 | 0.239 |
| IRI-C total | 12 | 2.280 | 0.023 |

*Notes. Effective n denotes the number of non-zero paired differences. Z was computed using the normal approximation to the Wilcoxon signed-rank test with tie correction based on absolute paired differences, and asymptotic p is the two-sided asymptotic p value derived from Z. Δ is defined as camp-exit minus camp-entry. Exact p values are reported in the main manuscript (Table 3).*

**Supplementary Table S8. Wilcoxon Z and asymptotic p values for Stroop outcomes**

| **Outcome** | **nₑff** | **Z** | **asymptotic p (two-sided)** |
| --- | --- | --- | --- |
| RT (game-related), ms | 12 | -2.353 | 0.019 |
| RT (neutral), ms | 12 | -2.040 | 0.041 |
| Accuracy (game-related) | 11 | 2.490 | 0.013 |
| Accuracy (neutral) | 12 | 2.824 | 0.005 |
| RT interference index | 12 | -0.784 | 0.433 |
| Accuracy interference index | 12 | -0.157 | 0.875 |

*Notes. Effective n denotes the number of non-zero paired differences. Z was computed using the normal approximation to the Wilcoxon signed-rank test with tie correction based on absolute paired differences, and asymptotic p is the two-sided asymptotic p value derived from Z. Δ is defined as camp-exit minus camp-entry. Exact p values are reported in the main manuscript (Table 4).*

**Supplementary Table S9. Counselor observational ratings across 12 sessions (n=12)**

| **Session** | **Cooperation willingness Mean±SD** | **Task completion quality Mean±SD** | **Engagement Mean±SD** | **Communication skills Mean±SD** | **Emotion regulation Mean±SD** | **Total score (0–100) Mean±SD** |
| --- | --- | --- | --- | --- | --- | --- |
| S1 | 5.38 ± 1.54 | 6.08 ± 1.36 | 5.25 ± 1.69 | 6.25 ± 2.35 | 6.42 ± 1.77 | 58.75 ± 13.65 |
| S2 | 5.88 ± 1.48 | 6.42 ± 1.28 | 5.83 ± 1.15 | 6.46 ± 2.04 | 6.79 ± 1.53 | 62.75 ± 10.96 |
| S3 | 6.29 ± 1.50 | 7.08 ± 1.49 | 6.29 ± 1.21 | 6.75 ± 1.82 | 7.00 ± 1.28 | 66.83 ± 11.07 |
| S4 | 6.33 ± 1.84 | 6.62 ± 1.43 | 6.46 ± 1.86 | 6.79 ± 1.83 | 7.04 ± 1.57 | 66.50 ± 12.75 |
| S5 | 6.83 ± 1.21 | 7.29 ± 1.18 | 6.79 ± 1.34 | 6.92 ± 1.87 | 7.38 ± 1.46 | 70.42 ± 10.83 |
| S6 | 7.00 ± 0.98 | 7.17 ± 0.86 | 6.83 ± 1.30 | 7.25 ± 1.75 | 7.25 ± 1.60 | 71.00 ± 8.69 |
| S7 | 6.92 ± 0.82 | 7.17 ± 0.83 | 7.04 ± 1.21 | 7.46 ± 1.51 | 7.38 ± 1.54 | 71.92 ± 6.47 |
| S8 | 7.08 ± 0.95 | 7.25 ± 0.89 | 7.38 ± 1.05 | 7.21 ± 1.57 | 7.21 ± 1.30 | 72.25 ± 7.97 |
| S9 | 6.88 ± 0.96 | 7.29 ± 0.86 | 7.38 ± 1.17 | 7.08 ± 1.40 | 7.29 ± 1.34 | 71.83 ± 6.74 |
| S10 | 7.33 ± 0.94 | 7.88 ± 0.71 | 7.46 ± 1.23 | 7.42 ± 1.56 | 7.33 ± 1.42 | 74.83 ± 8.00 |
| S11 | 7.58 ± 1.02 | 8.08 ± 0.63 | 7.67 ± 1.11 | 7.50 ± 1.45 | 7.46 ± 1.36 | 76.58 ± 7.74 |
| S12 | 7.50 ± 1.26 | 7.92 ± 1.18 | 7.38 ± 1.40 | 7.33 ± 1.72 | 7.25 ± 1.70 | 74.75 ± 11.66 |

*Notes. Values are mean±SD session-level ratings. Five domains were rated after each session. Each domain score ranges from 0 to 10 and was calculated as the mean of two subitems scored 0–10; the total score ranges from 0 to 100 and was calculated as the sum of all 10 subitems.*

**Supplementary Table S10. Participant satisfaction with camp sessions and logistics (n=12)**

| **Item** | **Mean ± SD** | **Median** | **High Satisfaction (≥4, %)** |
| --- | --- | --- | --- |
| Ice-breaking game | 4.00 ± 1.13 | 4.5 | 58.3 |
| "Painting" Conversation – Mandala Painting | 3.50 ± 1.57 | 3.5 | 50.0 |
| "Healing" Self – Mandala Flower Art | 3.58 ± 1.56 | 4.0 | 58.3 |
| Let's Exercise This Summer | 4.08 ± 1.08 | 4.5 | 66.7 |
| Embracing Inner Self | 3.25 ± 1.66 | 3.0 | 41.7 |
| Defeating Negative Emotions | 3.08 ± 1.62 | 3.0 | 33.3 |
| We Are All Protagonists (Mini Theater) | 3.50 ± 1.57 | 3.5 | 50.0 |
| There's a "Little Monster" Inside Us | 3.50 ± 1.57 | 3.5 | 50.0 |
| I'm So Lovable | 3.42 ± 1.56 | 3.0 | 41.7 |
| Youth Friendship | 3.75 ± 1.36 | 4.0 | 58.3 |
| "Wow", Invisible World | 3.08 ± 1.51 | 3.0 | 41.7 |
| We Are All Little Artists | 3.00 ± 1.41 | 2.5 | 33.3 |
| Activity Arrangement | 3.92 ± 1.16 | 4.0 | 66.7 |
| Organization and Management | 4.25 ± 0.97 | 4.5 | 83.3 |
| Camp Facilities and Environment | 4.25 ± 1.06 | 5.0 | 75.0 |
| Accommodation and Meals | 3.83 ± 0.94 | 4.0 | 83.3 |
| Support from Organizers | 4.33 ± 0.89 | 4.5 | 91.7 |
| Overall Experience | 4.17 ± 1.11 | 4.5 | 83.3 |

*Notes. Satisfaction ratings were collected at camp exit using a 1–5 Likert scale (1=lowest, 5=highest). Values are mean±SD across participants unless otherwise specified. Higher scores indicate greater satisfaction. Session titles and logistics items correspond to the prespecified post-camp evaluation form.*

**Supplementary Table S11. Spearman correlations between changes in primary outcomes and changes in other measures (exploratory, n=12)**

| **Variable** | **ΔGDSS ρ (p)** | **ΔGAS-7 ρ (p)** | **ΔVAS ρ (p)** |
| --- | --- | --- | --- |
| ΔGAS-7 (Post−Pre) | 0.750 (0.005) | — | 0.542 (0.069)† |
| ΔVAS (Post−Pre) | 0.394 (0.205) | 0.542 (0.069)† | — |
| ΔBIS-11 total (Post−Pre) | 0.280 (0.378) | 0.221 (0.490) | 0.505 (0.094)† |
| ΔConners (camp residential counselor–rated) total (Post−Pre) | 0.084 (0.794) | -0.205 (0.523) | 0.220 (0.491) |
| ΔSDS (standard score) (Post−Pre) | 0.456 (0.136) | 0.357 (0.255) | -0.005 (0.987) |
| ΔSAS (standard score) (Post−Pre) | 0.256 (0.422) | 0.222 (0.489) | -0.381 (0.222) |
| ΔSADS (Post−Pre) | 0.187 (0.561) | -0.191 (0.551) | -0.315 (0.318) |
| ΔIRI-C total (Post−Pre) | 0.193 (0.549) | 0.023 (0.943) | -0.675 (0.016) |
| Baseline PSQI global score | 0.250 (0.426) | 0.240 (0.447) | 0.767 (0.004) |
| Overall satisfaction (mean of 18 items) | 0.317 (0.316) | 0.104 (0.747) | -0.289 (0.361) |

*Notes. Δ=camp-exit score minus camp-entry score (Post−Pre). For symptom measures, more negative Δ indicates a larger reduction in symptoms; for IRI-C, more positive Δ indicates a larger increase in self-reported interpersonal responsiveness. PSQI was assessed at baseline only and was correlated directly with each Δ. All correlations are exploratory and unadjusted for multiple comparisons; p<0.05 indicates nominal significance and 0.05≤p<0.10 indicates a trend (†). “—” indicates not applicable.*

**Supplementary Table S12. Conceptual model and hypothesized mechanisms of change**

| **Theoretical component** | **Hypothesized maintaining process in adolescent IGD** | **Camp mechanism** | **Example program elements** |
| --- | --- | --- | --- |
| Cue-reactivity and craving framework | Gaming cues and habitual access to devices trigger craving and automatic gaming responses | Cue-context management and craving externalization | No smartphones or internet-enabled devices; supervised contact only; “Little Monster” craving activity |
| Cognitive–behavioral model of behavioral addiction | Maladaptive gaming-related cognitions, impaired self-monitoring, and difficulty interrupting gaming-related behavioral chains | Self-monitoring, trigger identification, coping rehearsal, and cognitive–behavioral reflection | Emotion-identification tasks; trigger mapping; pause-and-choose exercises; structured debriefing |
| Behavioral-control and delay-of-gratification principles | Loss of control, impulsive responding, poor task persistence, and difficulty delaying immediate reward | Rule-governed tasks, turn-taking, delayed responding, persistence training, and structured feedback | Mandala tasks; cooperative challenges; rule-based group games; task completion feedback |
| Reinforcement replacement / behavioral activation | Online gaming provides rapid reward, mastery, and achievement, while offline reward is relatively weak | Alternative offline reinforcement through mastery, movement, creativity, and peer recognition | Exercise session; art-based activities; team points; achievement log; peer feedback |
| Emotion-regulation model | Gaming is used to escape, suppress, or regulate negative affect | Emotion labeling, coping-response generation, and rehearsal of non-gaming regulation strategies | “Defeating Negative Emotions”; externalizing emotions/craving; coping-skills practice |
| Social-learning and interpersonal reinforcement | Reduced offline affiliation and social confidence increase reliance on gaming identity and online interaction | Cooperative peer interaction, role rehearsal, communication practice, and interpersonal feedback | Mini theater; youth friendship tasks; group cooperation; structured positive feedback |
| Relapse-prevention model | Return to the usual environment may reactivate gaming cues, craving, and habitual responses | High-risk situation identification and post-camp maintenance planning | Final quest; maintenance worksheet; coping scripts for high-risk situations |

*Note. This table presents the authors’ theory-guided mapping of the camp components to hypothesized mechanisms of change. It is intended to clarify the intervention rationale and the selection of outcome domains. The present single-group pilot evaluation was not designed to formally test mediation, isolate active ingredients, or determine the independent effect of any single component.*

**Supplementary Table S13. Assessment instruments, rationale, scoring, and interpretation.**

| **Assessment domain** | **Measure** | **Timing** | **No. of items** | **Response scale** | **Score range / scoring** | **Direction of interpretation** | **Criteria used in this study** | **Rationale for selection** | **Analytic role and interpretive boundary** |
| --- | --- | --- | --- | --- | --- | --- | --- | --- | --- |
| Core IGD symptom severity | Gaming Disorder Screening Scale, GDSS | Pre and Post | 18 | 1–4 Likert scale | Total score range 18–72 | Higher scores indicate greater gaming-disorder symptom severity | High-risk threshold: total score ≥47; severe threshold: total score ≥54; responder definition: ≥50% reduction in total score | Selected as a disorder-specific measure of core IGD symptom severity, directly aligned with cue-context management, craving-coping rehearsal, and non-gaming alternative reinforcement | Primary outcome. Interpreted as a short-term entry-to-exit signal rather than causal efficacy evidence without a control group or follow-up |
| Core gaming-addiction symptoms | Game Addiction Scale–7, GAS-7 | Pre and Post | 7 | 1–5 Likert scale | Total score range 7–35 | Higher scores indicate greater gaming-addiction symptom burden | Primary positivity rule: polythetic scoring, ≥4 of 7 items scored ≥3; sensitivity positivity rule: monothetic scoring, all 7 items scored ≥3 | Selected as a brief IGD-related symptom measure to complement GDSS and support comparability with prior gaming-disorder studies | Primary outcome. Interpreted as a short-term symptom signal; positivity transitions were descriptive |
| Craving | Visual Analog Scale, VAS craving | Pre and Post | 1 | 0–10 rating scale | Single-item score range 0–10 | Higher scores indicate stronger gaming craving | Moderate-to-severe craving: ≥5; low craving: ≤3; no craving: 0. Paired trajectory was described as decrease, no change, or increase | Selected as a proximal craving measure expected to be sensitive to cue reduction, craving externalization, and coping rehearsal | Primary outcome. Interpreted as short-term craving change; durability after return to usual environment was not assessed |
| Impulsivity / behavioral control | Barratt Impulsiveness Scale–11, Chinese version, BIS-11 | Pre and Post | 26 | 1–4 Likert scale | Total score range 26–104. The total score was used as the main BIS-11 outcome; subscale scores, if described, were calculated according to the source scoring key | Higher scores indicate greater impulsivity | High-impulsivity reference threshold: total score ≥68, reported descriptively | Selected because impaired control, delayed gratification, task persistence, and difficulty interrupting gaming-related response chains were central to the maintenance model and were targeted through rule adherence, delay, task persistence, and pause-and-choose exercises | Supportive secondary outcome. Interpreted cautiously as a short-term questionnaire-based shift, not as evidence of durable trait modification |
| Depressive symptoms | Zung Self-Rating Depression Scale, SDS | Pre and Post | 20 | 1–4 Likert scale | Raw score range 20–80; standard score = raw score × 1.25, rounded to integer | Higher standard scores indicate more severe depressive symptoms | Positive threshold: standard score ≥53; severe threshold: standard score ≥73 | Selected because gaming may function as emotion avoidance or negative-affect regulation, and the curriculum included emotion identification, coping rehearsal, and behavioral activation | Supportive secondary outcome. Interpreted as a domain-level signal, not as evidence that the camp treats depressive disorders |
| Anxiety symptoms | Zung Self-Rating Anxiety Scale, SAS | Pre and Post | 20 | 1–4 Likert scale | Raw score range 20–80; standard score = raw score × 1.25, rounded to integer | Higher standard scores indicate more severe anxiety symptoms | Positive threshold: standard score ≥50; moderate-to-severe threshold: standard score ≥60 | Selected because anxiety symptoms may contribute to gaming as avoidance or stress reduction, and emotion-regulation components were included in the curriculum | Supportive secondary outcome. Interpreted as a domain-level signal; the 7-day interval limits inference regarding sustained anxiety change |
| Social avoidance / social distress | Social Avoidance and Distress Scale, SADS | Pre and Post | 28 | Yes/no response format | Total score range 0–28; social avoidance and social distress subscale scores were calculated according to the source scoring key | Higher scores indicate greater social avoidance and distress | Reference thresholds: total score ≥17 and ≥19, reported descriptively only | Selected because reduced offline affiliation and social confidence may maintain reliance on gaming, whereas the camp targeted offline social participation through cooperation, role-play, peer interaction, and communication practice | Supportive secondary outcome. Interpreted cautiously over the 7-day interval; not used as a diagnostic social-anxiety assessment |
| Interpersonal responsiveness | Chinese version of the Interpersonal Reactivity Index, IRI-C | Pre and Post | 22 | 0–4 Likert scale | Total score range 0–88. Total and subscale scores were calculated according to the source scoring key | Higher total scores were interpreted cautiously as greater self-reported interpersonal responsiveness in this study; subscale-specific interpretation requires caution, particularly because higher personal distress reflects greater self-oriented distress | No clinical cutoff or responder definition was applied. Directional change was interpreted descriptively | Selected because group cooperation, perspective-taking, feedback, and role rehearsal were expected to engage interpersonal processes | Supportive secondary outcome. Interpreted as a broad interpersonal-responsiveness indicator, not as a clinical outcome with an established cutoff or as durable trait change |
| Observable behavioral dysregulation | Conners Teacher Rating Scale, 28-item version, completed by camp residential counselors | Pre and Post | 28 | 0–3 rating scale | Total score range 0–84; factor mean scores were calculated by dividing raw factor scores by the number of items. Factors included conduct problems, hyperactivity, inattention-passivity, and hyperactivity index | Higher scores indicate more frequent or severe observed behavioral problems | High ADHD-risk reference: Hyperactivity Index mean ≥1.5; abnormal reference: any factor mean ≥1.5. Both were reported descriptively only | Selected as a structured external observation of attention problems, impulsivity/hyperactivity, task persistence, and behavioral regulation in the residential camp context, complementing self-report outcomes and session-specific process ratings | Exploratory external-rating outcome. Context-bound observation only; not diagnostic ADHD screening and not equivalent to standard school-based teacher ratings |
| Baseline sleep quality | Pittsburgh Sleep Quality Index, PSQI | Baseline only | 19 self-rated items summarized into 7 component scores | Component scoring, each component 0–3 | Global score range 0–21, calculated as the sum of seven component scores | Higher scores indicate poorer sleep quality | Poor sleep quality: global score >5; moderate-to-severe sleep problems: global score >8. Baseline characterization only | Selected to characterize baseline sleep-related burden that may shape craving, executive control, affective regulation, daily routines, and response heterogeneity | Baseline contextual variable. Not assessed as a short-interval treatment outcome and excluded from the pre–post outcome set |
| Baseline family functioning | Family Adaptability and Cohesion Evaluation Scales II, Chinese Version, FACES II-CV | Baseline only | 30 | 1–5 Likert scale | Total score range 30–150; cohesion 16 items and adaptability 14 items were calculated separately for actual and ideal family functioning. Gap score was defined as Actual − Ideal | Higher actual scores indicate greater perceived family cohesion/adaptability; interpretation should consider the circumplex model and the Actual − Ideal discrepancy, and should not be simplified as “higher is always better.” More negative gap scores indicate perceived actual functioning further below the ideal | No clinical cutoff was applied. Actual, ideal, and Actual − Ideal gap scores were used for baseline characterization | Selected to characterize baseline family-context features that may influence adolescent IGD maintenance, daily routines, treatment engagement, and response heterogeneity | Baseline contextual variable. Not assessed as a short-interval treatment outcome or as evidence of family-function change |

*Note. This table summarizes the rationale, scoring, interpretation, and analytic role of each assessment instrument used in the present study. All reverse scoring had been completed before analysis, and analyses used the processed total scores, standard scores, component scores, or subscale scores specified above. In the source dataset, the GAS-7 variable was labelled as VGAS; in the manuscript, it is reported as GAS-7 for consistency with the cited scale terminology. Thresholds and cutoffs were used primarily for sample characterization, descriptive severity stratification, and responder analyses where applicable; unless explicitly stated, they should not be interpreted as diagnostic classifications. Given the single-group design, small sample, and 7-day interval, non-primary outcomes were interpreted as supportive or hypothesis-generating signals rather than as independent evidence of treatment benefit. For instruments that may partly reflect more stable individual characteristics, such as BIS-11 and IRI-C, entry-to-exit changes were interpreted as short-term questionnaire-based shifts rather than durable trait modification. For Conners, ratings were completed by camp residential counselors and were used as exploratory structured observations of behavioral dysregulation in the residential camp context, not as diagnostic ADHD screening or standard school-based teacher ratings. PSQI and FACES II-CV were baseline-only contextual measures and were not included in pre–post outcome analyses.*
